# A new LKB1 activator, piericidin analogue S14, retards renal fibrosis through promoting autophagy and mitochondrial homeostasis in renal tubular epithelial cells

**DOI:** 10.7150/thno.78376

**Published:** 2022-10-09

**Authors:** Canzhen Liu, Xiaoxu Wang, Xiaonan Wang, Yunfang Zhang, Wenjian Min, Ping Yu, Jinhua Miao, Weiwei Shen, Shuangqin Chen, Shan Zhou, Xiaolong Li, Ping Meng, Qinyu Wu, Fan Fan Hou, Youhua Liu, Peng Yang, Cheng Wang, Xu Lin, Lan Tang, Xuefeng Zhou, Lili Zhou

**Affiliations:** 1 Division of Nephrology, Nanfang Hospital, Southern Medical University; National Clinical Research Center for Kidney Disease; State Key Laboratory of Organ Failure Research; Guangdong Provincial Institute of Nephrology; Guangdong Provincial Key Laboratory of Renal Failure Research, Guangzhou, China.; 2 Department of Nephrology, Huadu District People's Hospital, Southern Medical University, Guangzhou, China.; 3 State Key Laboratory of Natural Medicines and Jiang Su Key Laboratory of Drug Design and Optimization, China Pharmaceutical University, Nanjing, China.; 4 Guangdong Provincial Key Laboratory of New Drug Screening, School of Pharmaceutical Sciences, Southern Medical University, Guangzhou, China.; 5 Division of nephrology, Department of medicine, the Fifth affiliated hospital of Sun Yat-Sen University, Zhuhai, Guangdong, China.; 6 Department of Nephrology, The Affiliated Hospital of Youjiang Medical University for Nationalities, Baise, Guangxi, China.; 7 CAS Key Laboratory of Tropical Marine Bio-resources and Ecology, Guangdong Key Laboratory of Marine Materia Medica, South China Sea Institute of Oceanology, Chinese Academy of Sciences, Guangzhou, China.

**Keywords:** LKB1, PA-S14, renal fibrosis, autophagy, AMPK

## Abstract

**Background:** Liver kinase B1 (LKB1) is the key regulator of energy metabolism and cell homeostasis. LKB1 dysfunction plays a key role in renal fibrosis. However, LKB1 activators are scarce in commercial nowadays. This study aims to discover a new drug molecule, piericidin analogue S14 (PA-S14), preventing renal fibrosis as a novel activator to LKB1.

**Methods:** Our group isolated PA-S14 from the broth culture of a marine-derived Streptomyces strain and identified its binding site. We adopted various CKD models or AKI-CKD model (5/6 nephrectomy, UUO, UIRI and adriamycin nephropathy models). TGF-β-stimulated renal tubular cell culture was also tested.

**Results:** We identified that PA-S14 binds with residue D176 in the kinase domain of LKB1, and then induces the activation of LKB1 through its phosphorylation and complex formation with MO25 and STRAD. As a result, PA-S14 promotes AMPK activation, triggers autophagosome maturation, and increases autophagic flux. PA-S14 inhibited tubular cell senescence and retarded fibrogenesis through activation of LKB1/AMPK signaling. Transcriptomics sequencing and mutation analysis further demonstrated our results.

**Conclusion:** PA-S14 is a novel leading compound of LKB1 activator. PA-S14 is a therapeutic potential to renal fibrosis through LKB1/AMPK-mediated autophagy and mitochondrial homeostasis pathways.

## Introduction

Chronic kidney disease (CKD) is becoming a global health problem 1, 2, which is at the high risk to develop into end-stage renal disease (ESRD) 3. Renal fibrosis is the common pathological feature for all kinds of CKD 3-5. However, there are no effective therapeutic strategies nowadays.

Kidney is in high metabolic need. The energy homeostasis plays a central role in maintaining normal kidney function 6, 7. Many reports have shown energy metabolism disorder highly contributes renal fibrosis 8-10. However, the modulator of energy metabolism remains unknown.

LKB1, also named STK11, is a serine/threonine kinase widely expressed in tissues 11-14. LKB1 is the key upstream kinase for activation of AMP-activated protein kinase (AMPK) 13, 15, a central regulator for metabolism 16, 17. AMPK is also highly involved in autophagy and mitochondrial homeostasis, the two crucial processes of cellular homeostasis and also key modulators in retarding renal fibrosis. The whole LKB1 protein contains C-terminal, N-terminal, and a central kinase domain (residues 49 to 309) 18-20. Several point site mutations in LKB1 kinase domain, such as D176, K78 and L67 residues, have been reported to be responsible for loss of function of LKB1, resulting in Peutz-Jeghers syndrome, an autosomal-dominant polyposis with multiple organ malignancies disorder 21. LKB1 dysfunction is also critical to renal tubular cell metabolic stress and fibrosis 22. However, LKB1 activator is not available in commercial nowadays. Hence, to develop a new LKB1 activator is of great importance to the therapeutic strategies in renal fibrosis.

Marine-derived microorganisms, especially marine-derived *Streptomyces* strains, have great potential for drug development 23. Recently, our group obtained 43 natural piericidins, including 29 new compounds, from two strains of marine-derived *Streptomyces* 24, 25. Among them, we screened a new compound, piericidin glycoside analogue S14 (PA-S14), which is an analogue of Piericidin A (PA), a well-known mitochondrial complex I inhibitor. Compared with PA, PA-S14 is at low toxicity and no inhibitory effects on complex I. Interestingly, we found PA-S14 serves as a new LKB1 activator, which effectively retards renal fibrosis through AMPK-mediated autophagy and mitochondrial homeostasis. The mechanism depends on its binding to D176 residue in LKB1 kinase domain. All these findings suggest PA-S14 is a promising therapeutic potential in renal fibrosis, and could be further developed its function on multiple organs as a novel LKB1 activator.

## Results

### LKB1/AMPK signaling is inhibited in a variety of experimental CKD models

**Figure 1:**
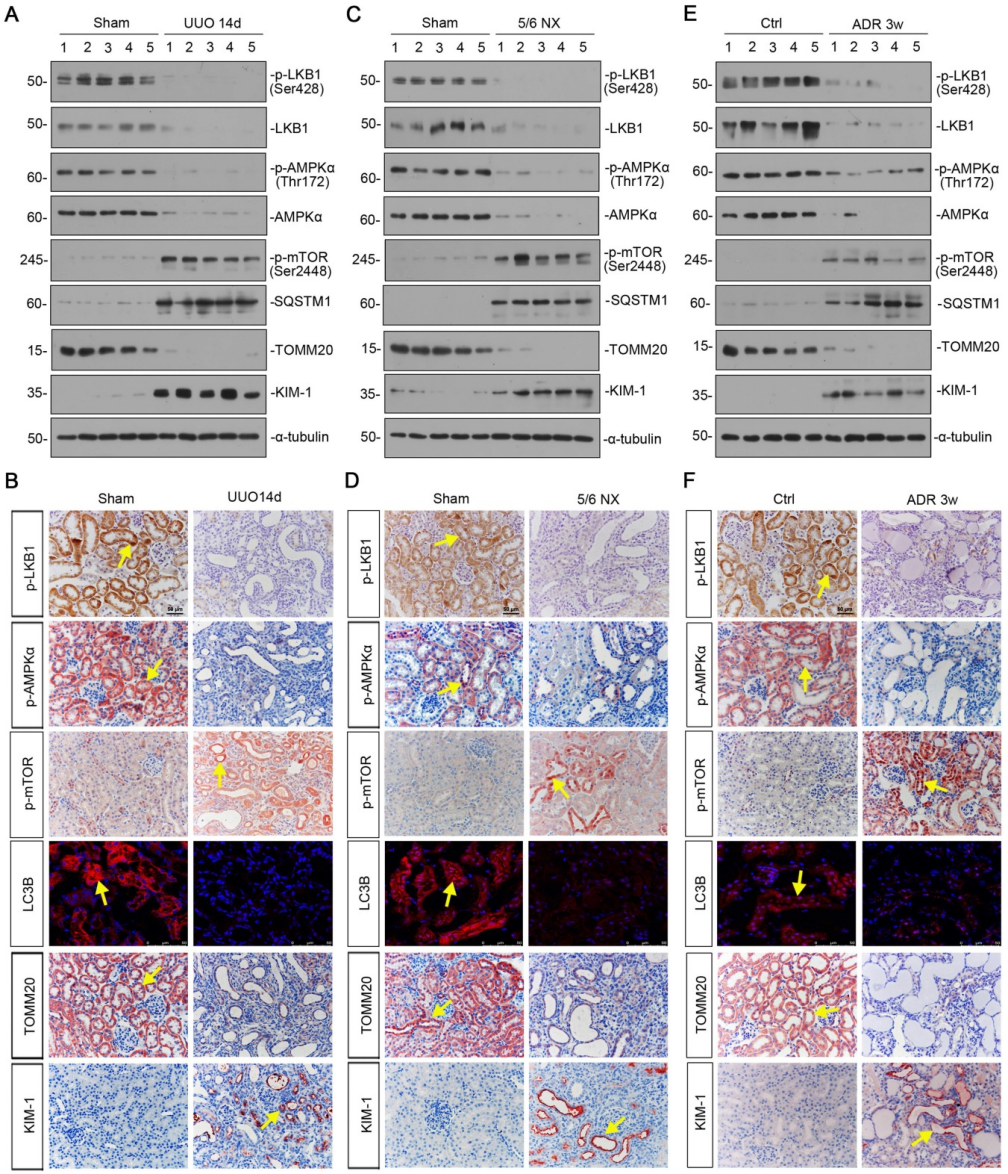
** LKB1-AMPK axis is inhibited in a variety of experimental CKD models.** (**A**) Representative western blotting showing renal expression of p-LKB1, LKB1, p-AMPKα, AMPKα, p-mTOR, SQSTM1, TOMM20, and KIM-1 in mice at 14 days after UUO. Numbers (1-5) indicate each individual animal in a given group. (**B**) Representative micrographs showing the expression of p-LKB1, p-AMPKα, p-mTOR, LC3B, TOMM20, and KIM-1 at 14 days after UUO. Paraffin or frozen kidney sections were stained with antibodies against p-LKB1, p-AMPKα, p-mTOR, LC3B, TOMM20, and KIM-1. Arrows indicate positive staining. Scale bar, 50 µm. (**C**) Representative western blot analyses showing renal expression of p-LKB1, LKB1, p-AMPKα, AMPKα, p-mTOR, SQSTM1, TOMM20, and KIM-1 protein in the remnant kidney in mice at 6 weeks after 5/6 nephrectomy. Numbers (1-5) indicate each individual animal in a given group. (**D**) Representative micrographs of immunostaining show renal expression of p-LKB1, p-AMPKα, p-mTOR, LC3B, TOMM20 and KIM-1 in mice at 6 weeks after 5/6 nephrectomy. Arrows indicate positive staining. Scale bar, 50 µm. (**E**) Representative western blotting analyses demonstrate the renal expression of p-LKB1, LKB1, p-AMPKα, AMPKα, p-mTOR, SQSTM1, TOMM20, and KIM-1 in mice at 3 weeks after the injection of adriamycin (ADR). Numbers (1-5) indicate each individual animal in a given group. (**F**) Representative micrographs show p-LKB1, p-AMPKα, p-mTOR, LC3B, TOMM20 and KIM-1 expression in kidneys in mice at 3 weeks after the injection of ADR. Arrows indicate positive staining. Scale bar, 50 µm.

We first examined the expression of LKB1/AMPK signaling in various CKD models, including unilateral ureteral obstruction (UUO), 5/6 nephrectomy (5/6NX), and adriamycin nephropathy (ADR). As shown in Figure 1A and B, and supplementary Figure S1, in UUO mice, the expressional levels of p-LKB1 and LKB1, p-AMPKα and AMPKα, were all significantly decreased. Meanwhile, the expression of p-mTOR and sequestosome 1 (SQSTM1/p62), the autophagosome-selective substrate, was upregulated accompanied by the downregulation of microtubule-associated protein 1A/1B-light chain 3 (LC3B), suggesting the autophagy deficiency. The expression of the outer mitochondrial membrane protein translocase of outer mitochondrial membrane 20 (TOMM20), a maker of mitochondrial mass, was found to be decreased with the increase in kidney injury molecule‐1 (KIM-1), a marker for tubular epithelial injury. The same results were also observed in 5/6NX and ADR models (Figures 1C to F, and S1). We also observed the decrease in AMPKα1 and increase in neutrophil gelatinase associated lipocalin (NGAL) and Fibronectin mRNA levels (Figure S1). These results demonstrate LKB1/AMPK axis is commonly suppressed in fibrotic kidneys.

### PA-S14 is a novel LKB1 activator through binding with D176 residue in kinase domain

**Figure 2:**
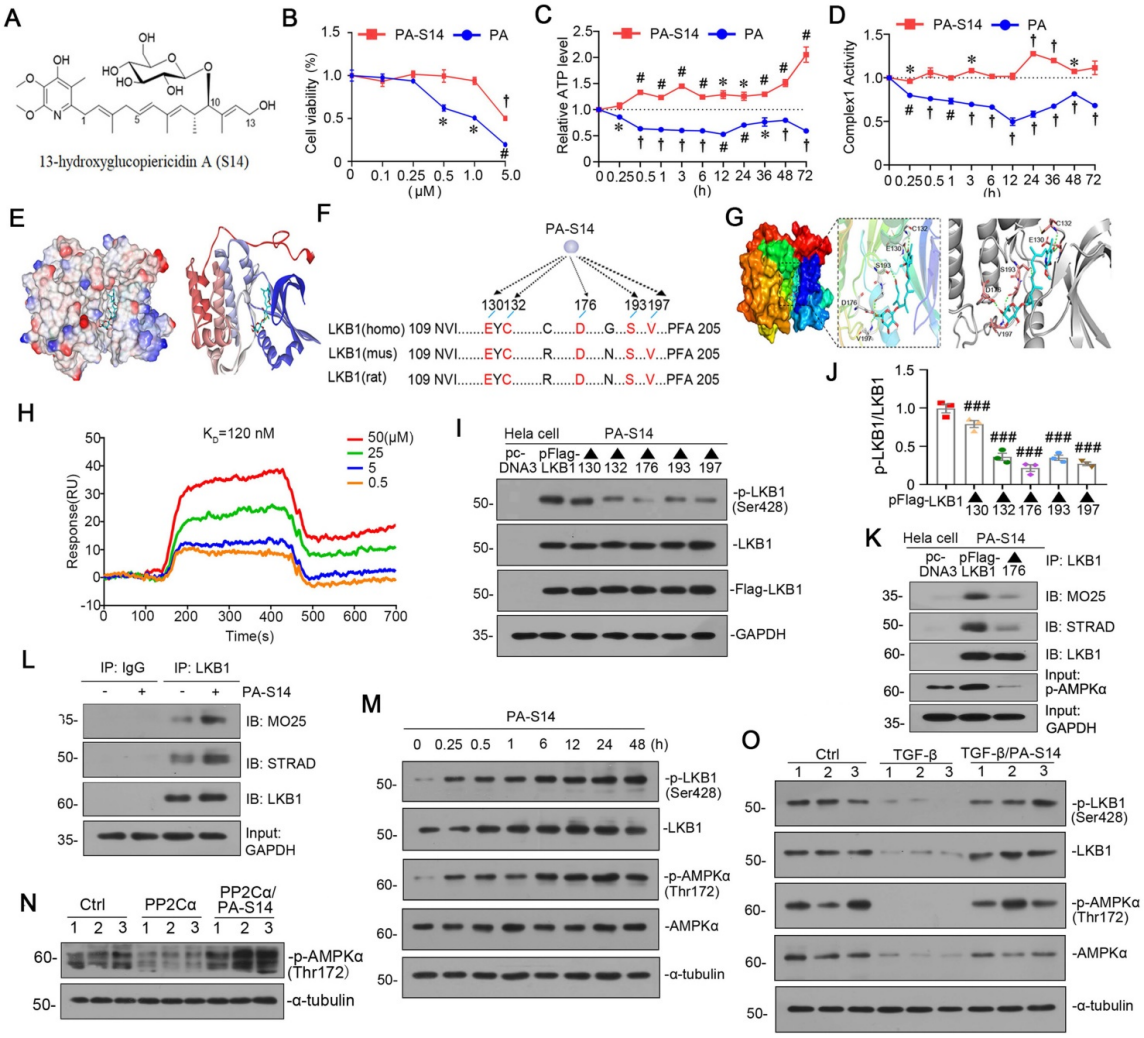
** PA-S14 is a novel LKB1 activator through binding with D176 residue in kinase domain.** (**A**) The detailed structural formula of PA-S14. (**B**) MTT assay in HKC‐8 cells treated with PA or PA-S14 at different concentrations. *P < 0.05, #*P* < 0.01, †*P* < 0.001 versus control group. (**C**) The ATP production in PA or PA-S14-treated HKC‐8 cells. *P < 0.05, #*P* < 0.01, †*P* < 0.001 versus control group. (**D**) Complex I activity was measured by spectrophotometrical assay in PA or PA-S14-treated HKC‐8 cells. *P < 0.05, #*P* < 0.01, †*P* < 0.001 versus control group. (**E**) The interaction binding mode diagram of LKB1 and PA-S14 was established based on the homology modeling technique in Discovery Studio 2021. The electrostatic surface of LKB1 is shown in blue, positively charged; red, negatively charged; white, neural residues. (**F, G**) Sequence analyses showed a high conservation sequence among human, mouse, and rat species. (**H**) The affinity of LKB1 to PA-S14 was shown. SPR analyses showed dose-dependent (0.5-50 µM) binding of PA-S14 to LKB1 on an immobilized sensor chip. The fitted constants are ka = 54.9M^-1^ S^-1^; kd = 6.57 × 10-6 S^-1^; KD = 1.2× 10^-7^ M. (**I-J**) Representative western blot and quantitative data showing the ratio of p-LKB1/LKB1. LKB1 kinase domain was mutated at five sites, i.e. E130, C132, D176, S193 and V197. ###*P* < 0.001 versus pFlag-LKB1 group. (**K**) Representative graphs showed D176 mutation (▲176) blocked the binding of LKB1 with MO25 or STRAD. Hela cells were transfected with pFlag-LKB1 or D176 mutated plasmid, and then treated with PA-S14 (0.5 µM) for 6 h. Co-immunoprecipitation was performed. Whole cell lysates were immunoprecipitated with an antibody against LKB1 and blotted with antibodies against MO25 and STRAD. (**L**) Representative graphs showing the binding of LKB1 with MO25 or STRAD. HKC-8 cells were treated with or without PA-S14 (0.5 µM) for 6 h. Whole cell lysates were immunoprecipitated with an antibody against LKB1 and blotted with antibodies against MO25 and STRAD. (**M**) HKC‐8 cells were treated with PA-S14 (0.5 µM) for indicated time period (0, 0.25, 0.5, 1, 6, 12, 24, 48 h). The expression levels of p-LKB1, LKB1, p-AMPKα, and AMPKα were then assessed by western blotting. (**N**) Representative western blot showing the expression of p-AMPKα. HKC-8 cells were pretreated with PA-S14 (0.5 µM) for 1 h, followed by the stimulation of PP2Cα for 24 h. (**O**) Representative western blot showing protein expression of p-LKB1, LKB1, p-AMPKα, and AMPKα in different groups. HKC-8 cells were pretreated with PA-S14 (0.5 µM) for 1 h, followed by the stimulation of TGF-β1 (5 ng/ml) for 24 h. Numbers (1-3) indicate each individual culture in a group.

PA-S14 (Figures 2A, S2), which was recently obtained by our group, is a glycosylated and hydroxylative derivative of piericidin A (PA). Compared to PA, PA-S14 only had a low-level toxicity at 5 μM, whereas PA exhibited a high toxicity at 0.5 μM (Figure 2B). Adenosine triphosphate (ATP) production and complex I activity were next assessed. Compared with the continuous inhibition of PA on ATP production and complex I activity, PA-S14 showed no inhibitory effects but increased their activities (Figure 2C and D). These prompted us to investigate whether PA-S14 has other properties different from PA.

Notably, LKB1 is the central controller to energy metabolism. We first performed molecular docking of PA-S14 with LKB1 using LibDock program (in Discovery studio 2021). As shown, PA-S14 has a perfect binding activity with LKB1 kinase domain on residues E130, C132, D176, S193 and V197, all of them are highly conserved (Figure 2E to G). To testify the binding specificity of PA-S14 with LKB1, we performed surface plasmon resonance (SPR), a method to measure the kinetics and amplitude of protein/compound and protein binding. As shown in Figure 2H, PA-S14 directly bound to LKB1, and their binding affinity was strong with equilibrium dissociation constant (KD) calculated as 120 nM. We then identified the key residue of LKB1 responsible for PA-S14 binding in Hela cells, a cell line without endogenous LKB1 expression. Hela cells were transfected with the Flag-tagged LKB1 wild type or mutated plasmids, and then stimulated with PA-S14. As shown, PA-S14 induced the phosphorylated activation of LKB1 in the wild type plasmid-transfected cells, however, the mutation on E130, C132, D176, S193 and V197 significantly blocked it. Especially D176 mutation (▲176) showed the strongest blocking effect (Figure 2I and J). The complex formation of LKB1, MO25 and STRAD, an important process that functionally facilitates AMPK activation, was also checked. As shown in Figure 2K, PA-S14 treatment greatly triggered the complex formation in Hela cells in Flag-tagged LKB1 wild type plasmid-transfected group, but not in D176 mutation group.

We next examined the effects of PA-S14 on LKB1/AMPK signaling in renal tubular cells. As shown, PA-S14 induced an increase in the complex formation of MO25, STRAD and LKB1 (Figure 2L). Furthermore, PA-S14 had a quick and persistent activation activity on p-LKB1 and p-AMPKα (Figure 2M). Of note, PA could not induce LKB1 activation, and only had a very low activating activity on AMPKα at a very small dose (Figure S3). The PP2Ca is the member of PP2C family phosphatase which could inhibit AMPK activity. As shown, PP2Cα's inhibitory effect on p-AMPKα was greatly blocked by co-treatment with PA-S14 (Figure 2N). The stimulatory effects of PA-S14 on LKB1/AMPK signaling were also demonstrated in cultured renal tubular cells treated with TGF-β (Figure 2O).

### PA-S14 promotes autophagic flux and mitochondrial biogenesis through LKB1 signaling *in vitro*

**Figure 3:**
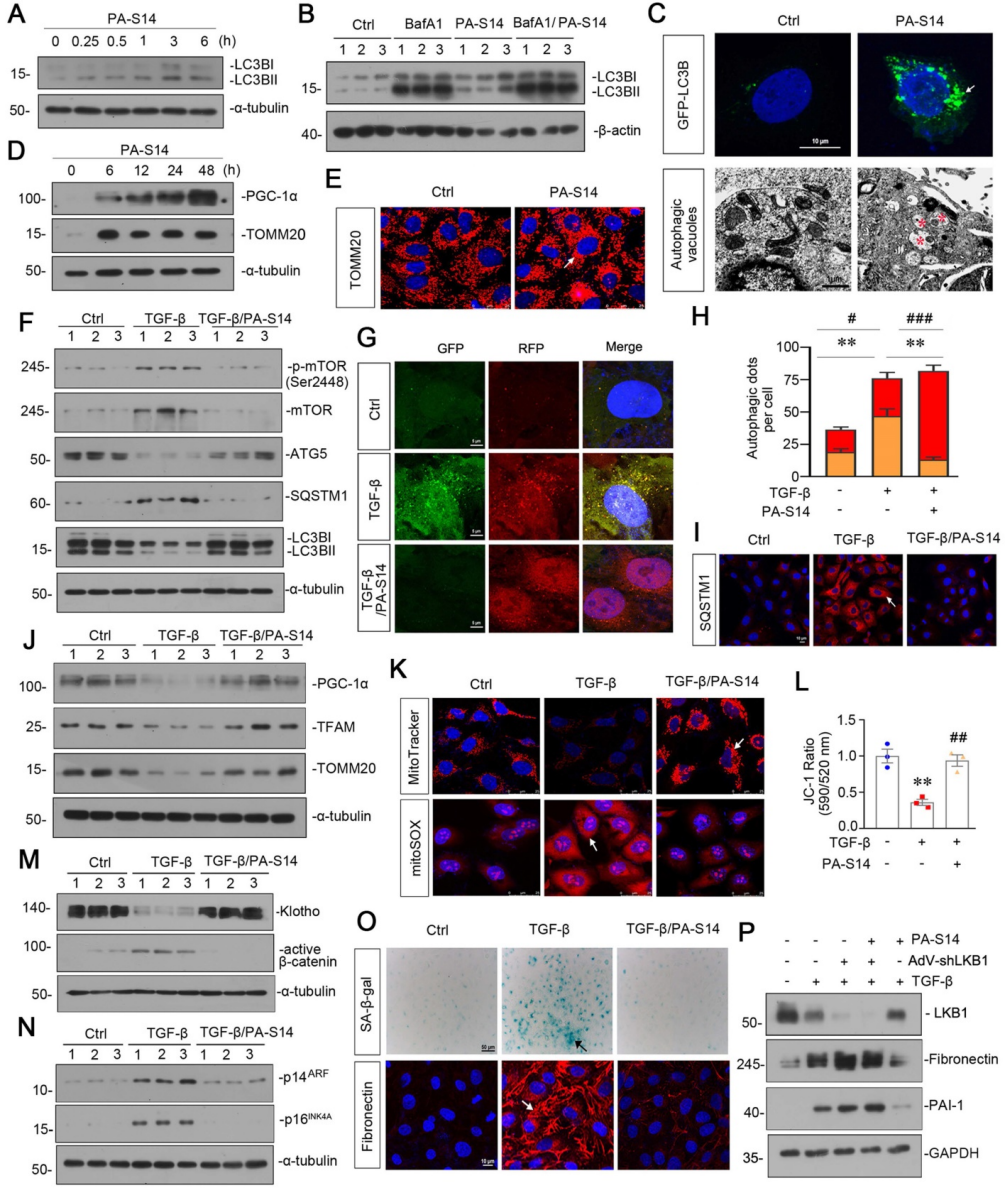
**PA-S14 promotes autophagic flux and mitochondrial biogenesis through LKB1 signaling *in vitro*.** (**A**) HKC‐8 cells underwent PA-S14 (0.5 µM) treatment for the indicated time period (0, 0.25, 0.5, 1, 3, 6 h). The expressional level of LC3BII/LC3BI ratio was then detected by western blotting. (**B**) HKC-8 cells were pretreated with PA-S14 (0.5 µM) for 1 h, followed by the stimulation of 100 nM of bafilomycin A1 (Baf A1) for 6 h. LC3BII/I ratio was analyzed by western blotting. (**C**) Representative micrographs showed that PA-S14 enhanced LC3BII punctae formation. HKC-8 cells were transiently transfected with GFP-LC3B plasmid for 24 h and were then stimulated with or without PA-S14 (0.5 µM) for another 24 h. The cells were observed by fluorescence microscopy. Arrows indicate positive staining. Scale bar, 10 µm. Representative electron microscopy (TEM) micrographs showed that PA-S14 increased autophagic vacuoles (red asterisks). HKC-8 cells were treated with PA-S14 (0.5 µM) for 24 h. Scale bar, 1 µm. (**D**) HKC‐8 cells were treated with PA-S14 (0.5 µM) for indicated time period (0, 6, 12, 24, 48 h). The expression levels of PGC-1α and TOMM20 were then assessed by western blotting. (**E**) Representative micrographs showing the immunofluorescence staining of TOMM20. HKC‐8 cells were treated with PA-S14 (0.5 µM) for 24 h. Arrows indicate positive staining. Scale bar, 25 µm. (**F**) Representative western blot showing the expression of the LC3BⅡ/Ⅰ ratio, SQSTM1, ATG5, p-mTOR, and mTOR in different groups. HKC‐8 cells were pretreated with PA-S14 (0.5 µM) for 1 h, and then treated with TGF-β1 (5 ng/ml) for 24 h. Numbers (1-3) indicate each individual culture in a group (n = 3). (**G**) Representative micrographs show that PA-S14 promoted autophagic flux. HKC-8 cells were pretransfected with lentivirus expressing RFP-GFP-LC3B for 24 h and were then stimulated by TGF-β1 (5 ng/ml) for 24 h with or without PA-S14 (0.5 µM). Natural-pH LC3B-positive autophagosomes (green fluorescence) and acidic-pH LC3B-positive autophagolysosomes (red fluorescence) were assessed. (**H**) Natural-pH LC3B-positive autophagosomes (green fluorescence) and acidic-pH LC3B-positive autophagolysosomes (red fluorescence) were assessed. Quantitative data of autophagosomes (gold) and autophagolysosomes (red) for Figure 3G. **P < 0.01 versus TGF-β1 alone (autophagosomes); #*P* < 0.05, ###*P* < 0.001 versus TGF-β1 alone (autophagolysosomes) (n = 10). (**I**) Representative fluorescence micrographs show SQSTM1 staining. HKC‐8 cells were pretreated with PA-S14 (0.5 µm) for 1 h and then treated with TGF-β1 (5 ng/ml) for 12 h. Cells were stained with an antibody against SQSTM1 (red) and counterstained with DAPI (blue). Arrows indicate positive staining. Scale bar, 10 µm. (**J**) Representative western blot showing the expression of PGC‐1α, TFAM and TOMM20. HKC‐8 cells were treated with TGF-β1 (5 ng/ml) alone or cotreated with PA-S14 (0.5 µM) for 24 h. Numbers (1-3) indicate each individual culture in a group. (**K**) Representative MitoTracker and mitoSOX staining micrographs show the mitochondrial mass and ROS production. After pretreatment with PA-S14 (0.5 µM) for 1 h, HKC‐8 cells were treated with TGF-β1 (5 ng/ml) for 24 h. Arrows indicate positive staining. Scale bar, 25 µm. (**L**) Graphical representation of mitochondrial membrane potential (MMP). MMP was detected by JC‐1 staining and analyzed by flow cytometry. After pretreatment with PA-S14 (0.5 µM) for 1 h, HKC‐8 cells were treated with TGF-β1 (5 ng/ml) for 12 h and then stained with JC‐1 dye. The MMP is shown as the ratio of the fluorescence intensity at absorbance of 590 nm (JC‐1 aggregate) to 520 nm (JC‐1 monomer). **P < 0.01 versus control group; ##*P* < 0.01 versus TGF-β1 group. (**M, N**) Representative western blot showing the expression of Klotho and active β-catenin. HKC‐8 cells were pretreated with PA-S14 (0.5 µM) for 1 h and then treated with TGF-β1 (5 ng/ml) for 24 h. Representative western blot showing the expression of p14^ARF^ and p16^INK4A^ in different groups. HKC‐8 cells were pretreated with PA-S14 (0.5 µM) for 1 h and then treated with TGF-β1 (5 ng/ml) for 60 h. Numbers (1-3) indicate each individual culture in a group. (**O**) Representative micrographs show SA‐β‐gal activity staining and the immunofluorescence staining of Fibronectin. After pretreatment with PA-S14 (0.5 µM) for 1 h, HKC‐8 cells were treated with TGF-β1 (5 ng/ml) for 60 h to test SA‐β‐gal activity. Arrows indicate positive staining. Scale bar, 50 µm. HKC‐8 cells were pretreated with PA-S14 (0.5 µM) for 1 h and then treated with TGF-β1 (5 ng/ml) for 24 h to test Fibronectin expression. Arrows indicate positive staining. Scale bar, 10 µm. (**P**) Representative western blot showing the expression of Fibronectin and PAI-1 in different groups. HKC-8 cells were infected with adenovirus expressing LKB1 shRNA (sh-LKB1), followed by pretreatment with PA-S14 (0.5 µM) for 1 h, and then treated with TGF-β1 (5 ng/ml) for another 24 h.

We then tested the effects of PA-S14 on autophagy and mitochondrial biogenesis. As shown, PA-S14 induced the formation of LC3B II, a marker of autophagosome maturation (Figure 3A), even if under the stimulation condition of bafilomycin (BafA1), an inhibitor of autophagosome-lysosome fusion (Figure 3B). The fluorescence of GFP-LC3B and electron microscopy assessment also demonstrated the formation of LC3B punctae and autophagic vacuole formation in PA-S14-treated cells (Figure 3C). Furthermore, PA-S14 promoted the expression of PGC-1α and TOMM20, the two markers of mitochondrial biogenesis (Figure 3D and E).

In TGF-β-treated cells, co-treatment with PA-S14 inhibited the expression of p-mTOR, mTOR, SQSTM1, and restored the expression of ATG5 and LC3BII formation (Figure 3F). We next assessed autophagic flux. The RFP-GFP-LC3B sensor enables LC3B-positive, neutral-pH autophagosomes as green fluorescence, whereas LC3B-positive acidic-pH autophagolysosomes exhibit red fluorescence. As shown, TGF-β induced autophagosome accumulation without fusion with lysosomes, as indicated by their golden color (overlap of red and green fluorescence), however, co-treatment with PA-S14 triggered autophagic flux as shown by red puncta due to autophagosomes fusion with lysosomes (Figure 3G and H). Consistently, the expression of SQSTM1 was also greatly decreased in PA-S14 co-treated cells (Figure 3I). PA-S14 also restored the expression of PGC-1α, TFAM and TOMM20, the mitochondrial biogenesis markers, in TGF-β-treated cells (Figure 3J). MitoTracker, mitoSOX, and JC-1 staining also showed PA-S14 greatly preserved mitochondrial mass, inhibited mitochondrial ROS production, and preserved the mitochondrial membrane potential (Figure 3K and L). We also observed PA-S14 restored the expression of Klotho, and inhibited the expression of active β-catenin, p14^ARF^, and p16^INK4A^, the cellular senescence markers (Figure 3M and N). SA‐β‐gal activity and Fibronectin staining also showed co-treatment with PA-S14 inhibited cellular senescence and fibrogenesis (Figure 3O). LKB1 was then ablated by an adenovirus expressing LKB1 shRNA (sh-LKB1). Of interest, PA-S14 showed no effects in TGF-β-treated cells when LKB1 was ablated, further suggesting the specific active activity of PA-S14 on LKB1 (Figure 3P).

### PA-S14 induces autophagy and mitochondrial biogenesis in healthy mice through activating LKB1/AMPK signaling

**Figure 4:**
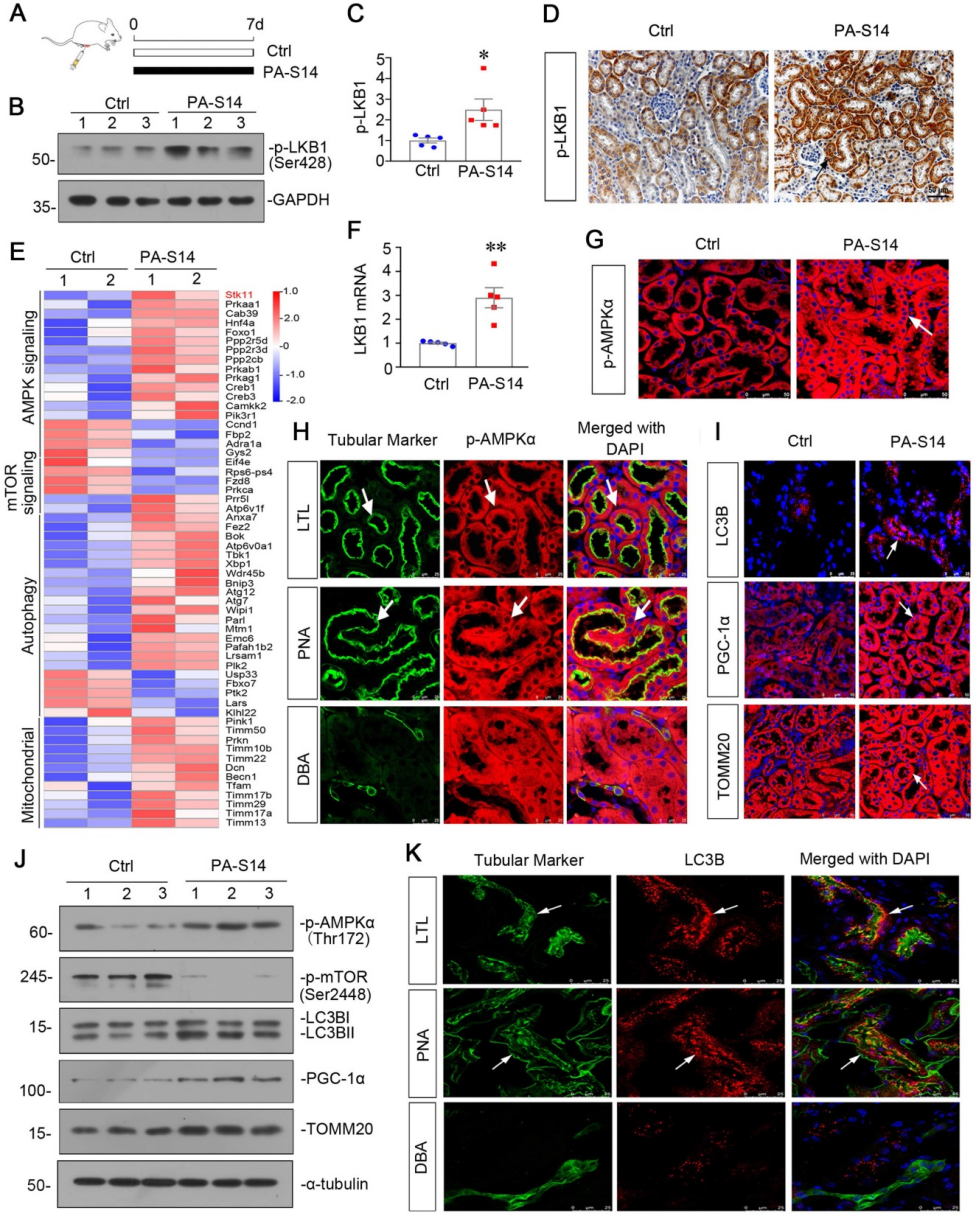
** PA-S14 induces autophagy and mitochondrial biogenesis in healthy mice through LKB1/AMPK signaling**. (**A**) Diagram shows the experimental design. Black line indicates PA-S14 treatment (1.0 mg/kg body weight). (**B-C**) Representative western blot and quantitative data showing renal expression of p-LKB1. Numbers (1-3) indicate each individual animal in a given group. *P < 0.05 versus control mice (n = 5). (**D**) Representative micrographs show renal expression of p-LKB1. Paraffin kidney sections were immunostained with an antibody against p-LKB1. Arrow indicates positive staining. Scale bar, 50 µm. (**E**) Representative heatmap gene expression of RNA sequencing analysis show several important pathways involving AMPK signaling, mTOR signaling, autophagy and mitochondria. (**F**) Graphic presentation shows the relative levels of renal expression of LKB1 mRNA in two groups as indicated. **P < 0.01 versus control mice (n = 5). (**G**) Representative micrographs show renal expression of p-AMPKα. Frozen kidney sections were stained for p-AMPKα. Arrows indicate positive staining. Scale bar, 50 µm. (**H**) Double immunofluorescence staining demonstrates the expression of p‐AMPKα predominantly in the proximal tubular and distal tubular epithelium. Kidney sections were co-stained for p‐AMPKα (Red) and various segment-specific tubular markers (green), respectively. Segment-specific tubular markers were used as follows: lotus tetragonolobus lectin (LTL); distal tubule, peanut agglutinin (PNA); and collecting duct, dolichos biflorus agglutinin (DBA). Arrows indicate positive tubules with colocalization of p-AMPKα and specific tubular markers. Scale bar, 25 µm. (**I**) Representative micrographs show renal expression of LC3B, PGC-1α, and TOMM20. Frozen kidney sections were stained for LC3B, PGC-1α, and TOMM20. Arrows indicate positive staining. Scale bar, 25 or 50 µm. (**J**) Representative western blot showing renal expression of p-AMPKα, p-mTOR, LC3B, PGC-1α, and TOMM20. Numbers (1-3) indicate each individual animal in a given group. (**K**) Double immunofluorescence staining demonstrates the expression of LC3B predominantly in the proximal tubular and distal tubular epithelium. Scale bar, 25 µm.

We first treated healthy mice with PA-S14 (Figure 4A). As shown, PA-S14 induced the activation of p-LKB1, which was primarily expressed in renal tubules (Figure 4B to D). We then performed the transcriptomics sequencing analysis. Heatmap analysis of the expression of transcripts showed that LKB1 (STK11) /AMPK signaling, autophagy and mitochondrial biogenesis activity were activated, while mTOR signaling was downregulated (Figure 4E). The upregulation of LKB1 mRNA was testified by qPCR (Figure 4F). Immunofluorescence of p-AMPKα staining showed PA-S14 induced its upregulation in tubules (Figure 4G). We then performed the costaining of p-AMPKα and different segments of renal tubules in PA-S14-treated mice. As shown, p-AMPKα upregulation was primarily co-localized with lotus tetragonolobus lectin (LTL), a marker of proximal tubules, and peanut agglutinin (PNA), a marker of distal convoluted tubules, but weakly co-expressed with dolichos biflorus agglutinin (DBA), a marker of collecting duct epithelium (Figure 4H). PA-S14 also induced the increase in LC3B punctae formation, PGC-1α and TOMM20 expression (Figure 4I). We also performed western blotting analysis. As shown, the expressional levels of p-AMPKα, PGC-1α, and TOMM20 were induced by PA-S14. Moreover, PA-S14 induced the transformation of LC3BI to LC3BII (Figure 4J), immunofluorescence also confirmed that LC3B punctae trigged by PA-S14 was primarily located in proximal and distal tubules (Figure 4K). We also observed the effects of PA-S14 on serum biochemical indexes and its influence to several important organs. As shown, there was no difference between control and PA-S14-treated group (Figure S4).

### PA-S14 promotes autophagy and preserves mitochondrial biogenesis in UIRI mice via activating LKB1/AMPK signaling

**Figure 5:**
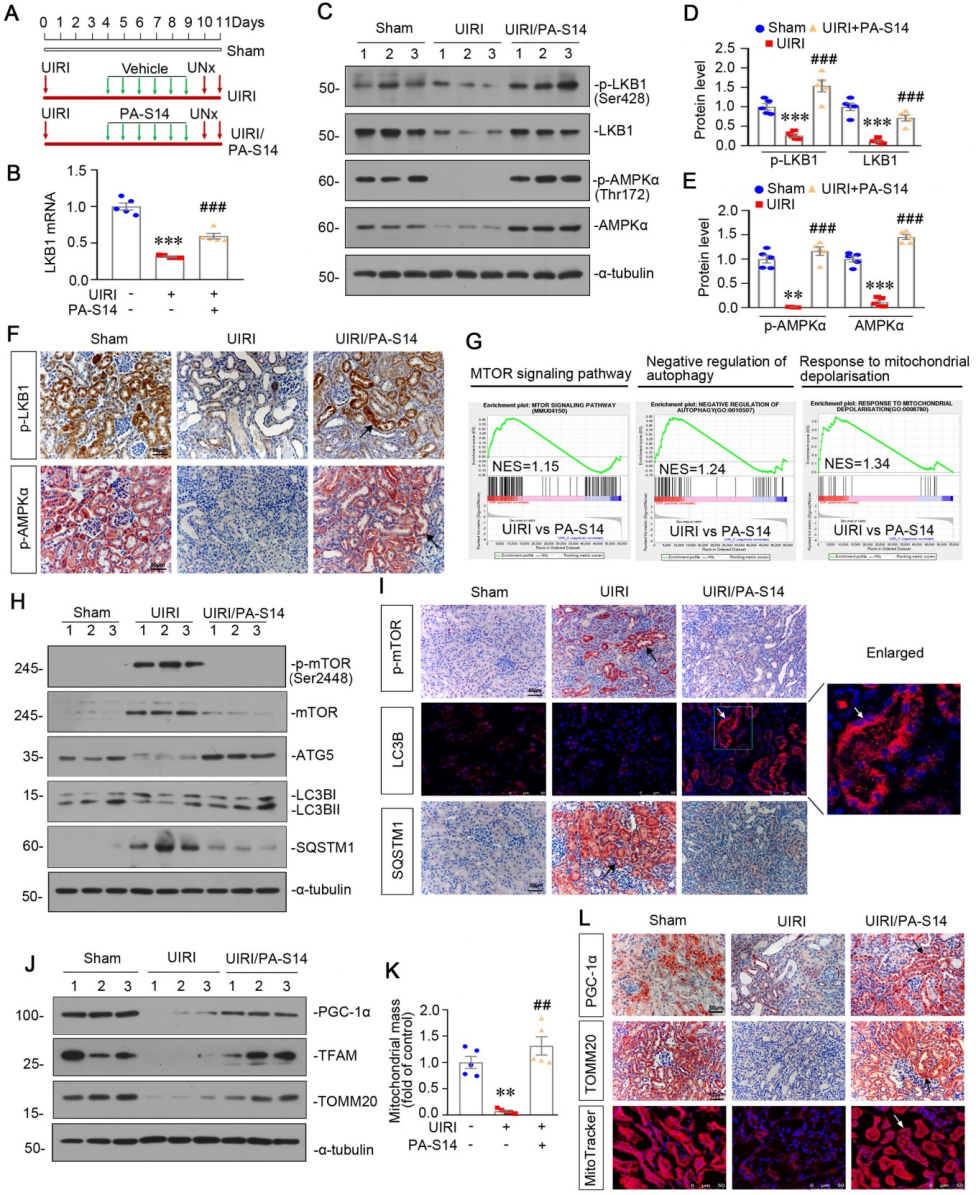
** PA-S14 promotes autophagy and mitochondrial biogenesis in UIRI mice via activation of LKB1-AMPK signaling.** (**A**) Diagram shows the experimental design. Red arrows indicate the time points undergoing UIRI, UNx (uni-nephrectomy) and sacrifice, respectively. Green arrows indicate PA-S14 treatment (1.0 mg/kg body weight). (**B**) Quantitative PCR result showing renal expression of LKB1 in different groups as indicated. ***P < 0.001 versus sham controls; ###*P* < 0.001 versus UIRI (n = 5). (**C-E**) Representative western blot and quantitative data showing renal expression of p-LKB1, LKB1, p-AMPKα, and AMPKα in different groups. Numbers (1-3) indicate each individual animal in a given group. **P < 0.01, ***P < 0.001 versus sham controls; ###*P* < 0.001 versus UIRI (n = 5). (**F**) Representative micrographs show the p-LKB1 and p-AMPKα expression in different groups as indicated. Kidney sections were stained with antibodies against p-LKB1 and p-AMPKα, respectively. Arrows indicate positive staining. Scale bar, 50 µm. (**G**) GSEA enrichment analysis showing that mTOR signaling pathway, negative regulation of autophagy and response to mitochondrial depolarization were enriched in UIRI. NES, normalized enrichment score; Nominal p-value < 0.05 or FDR q-value < 0.25. (**H**) Representative western blot showing renal expression of the p-mTOR, mTOR, ATG5, LC3BII/I ratio and SQSTM1 in different groups. Numbers (1-3) indicate each individual animal in a given group. (**I**) Representative micrographs show renal expression of p-mTOR, LC3B and SQSTM1. Paraffin kidney sections were stained with antibodies against p-mTOR and SQSTM1. The frozen kidney sections were stained with an antibody against LC3B. Arrows indicate positive staining. Scale bar, 50 µm. (**J**) Western blotting analyses show renal expression of PGC‐1α, TFAM and TOMM20 in different groups. Numbers (1-3) represent different individual animals in a given group. (**K**) Quantification of renal mitochondrial mass is shown. Mitochondrial mass was determined by the fluorescence intensity of MitoTracker deep red staining normalized to DAPI. **P < 0.01 versus sham controls; ##*P* < 0.01 versus UIRI (n = 5). (**L**) Representative micrographs show the expression of PGC-1α, TOMM20 and mitochondrial mass. Paraffin kidney sections were stained with antibodies against PGC-1α and TOMM20. The frozen kidney sections were stained with MitoTracker deep red (3 µm) probe. DAPI (4′,6‐diamidino‐2‐phenylindole) was used to stain the nuclei (blue). Arrows indicate positive staining. Scale bar, 50 µm.

We then tested the effects of PA-S14 in unilateral ischemia-reperfusion (UIRI) model, an AKI-CKD model 26 (Figure 5A). LKB1/AMPK signaling was first examined. As shown, PA-S14 significantly preserved the mRNA expression of LKB1 (Figure 5B), and restored the expression of p-LKB1, LKB1, p-AMPKα and AMPKα proteins in UIRI mice (Figure 5C to E). Similar results were observed when p-LKB1 and p-AMPKα were tested by immunostaining (Figure 5F). We next performed transcriptsome sequencing. Gene Set Enrichment Analysis (GSEA) revealed PA-S14 downregulated mTOR signaling, promoted autophagy, and inhibited mitochondrial depolarization (Figure 5G). Hence, we next analyzed autophagy and mitochondrial biogenesis. As shown, PA-S14 inhibited the expression of p-mTOR, mTOR, and SQSTM1, and triggered the upregulation of ATG5 and LC3BII formation (Figures 5H, S5). Simialr results were observed when p-mTOR, LC3B and SQSTM1 were stained by immunofluorescence and immunohistochemistry (Figure 5I). Furthermore, PA-S14 restored the expression of PGC-1α, TFAM, and TOMM20 expression (Figures 5J, S5). Mitochondrial mass quantification and immunostaining results also revealed PA-S14 greatly restored mitochondrial biogenesis activity (Figure 5K and L).

### PA-S14 protects against tubular cell senescence and renal fibrosis in UIRI mice

**Figure 6:**
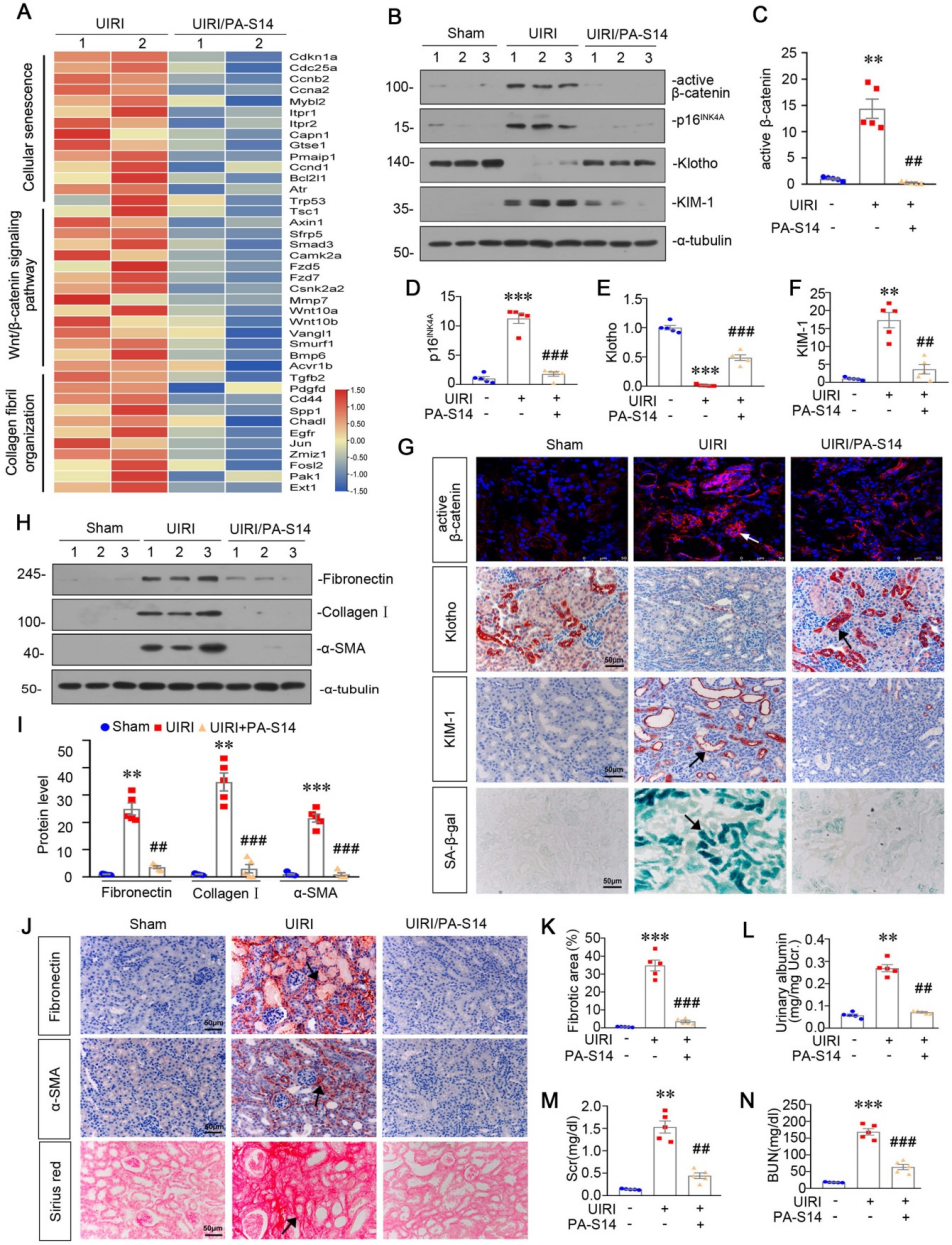
** PA-S14 protects against renal fibrosis and tubular cell senescence in UIRI mice.** (**A**) The heatmap, generated from the RNA-seq analysis, showing cellular senescence, Wnt/β-catenin signaling pathway and collagen fibril organization gene expression in UIRI and UIRI/PA-S14 groups. (**B-F**) Representative western blot and quantitative data showing renal expression of active-β-catenin, p16^INK4A^, Klotho and KIM-1. Numbers (1-3) indicate each individual animal in a given group. **P < 0.01, ***P < 0.001 versus sham controls; ##*P* < 0.01, ^###^*P* < 0.001 versus UIRI (n = 5). (**G**) Representative micrographs show the expression of active β-catenin, Klotho, KIM-1, and SA‐β‐gal activity in different groups as indicated. Frozen kidney sections were stained for active β-catenin expression and SA‐β‐gal activity. Paraffin sections were stained with antibodies against Klotho and KIM-1, respectively. Arrows indicate positive staining. Scale bar, 50 µm. (**H-I**) Representative western blot and quantitative data show the reduction of renal Fibronectin, Collagen I, and α‐SMA expression after PA-S14 treatment. Numbers (1-3) indicate each individual animal in given group. **P < 0.01, ***P < 0.001 versus sham controls; ##*P* < 0.01, ###*P* < 0.001 versus UIRI (n = 5). (**J**) Representative staining micrographs show Sirius red staining, and renal expression of Fibronectin and α‐SMA. Paraffin sections were performed to Sirius red staining or immunostained with antibodies against Fibronectin and α‐SMA. Arrows indicate positive staining. Scale bar, 50 µm. (**K**) Graphical representations of the degree of fibrotic lesions in different groups after quantitative determination of Sirius red staining intensity. ***P < 0.001 versus sham controls; ###*P* < 0.001 versus UIRI (n = 5). (**L**) Urinary albumin levels in different groups. Urinary albumin was expressed as milligrams per milligram creatinine. **P < 0.01 versus sham controls; ##*P* < 0.01 versus UIRI (n = 5). (**M-N**) The treatment of PA-S14 significantly decreased serum creatinine and BUN levels. **P < 0.01, ***P < 0.001 versus sham controls; ##*P* < 0.01, ###*P* < 0.001 versus UIRI (n = 5).

From the transcriptsome sequencing, we found PA-S14 had strong inhibitory effects on cellular senescence, Wnt/β-catenin signaling, and collagen fibril organization (Figure 6A). We then testified these pathways. As shown, PA-S14 decreased the expression of active β-catenin, p16^INK4A^, and KIM-1, but restored Klotho expression, suggesting its inhibitory effects on cellular senescence (Figure 6B to F). Similar results were observed when active β-catenin, Klotho and KIM-1 were assessed by immunostaining. Moreover, SA‐β‐gal activity staining also revealed PA-S14 greatly inhibited cellular senescence (Figure 6G). Furthermore, PA-S14 strongly blocked the upregulation of fibronectin, collagen I and α‐SMA, the firbogenesis markers (Figure 6H and I). Similar results were observed when fibronectin and α‐SMA were stained by immunohistochemistry (Figure 6J). Sirius red staining and its quantification also revealed PA-S14 strongly retarded renal fibrosis (Figure 6J and K). We also found PA-S14 greatly decreased urinary albumin, serum creatinine (Scr) and blood urea nitrogen (BUN) secretion, suggesting its protective role in renal function (Figure 6L to N).

### PA-S14 ameliorates renal fibrosis and tubular cell senescence in adriamycin nephropathy mice

**Figure 7:**
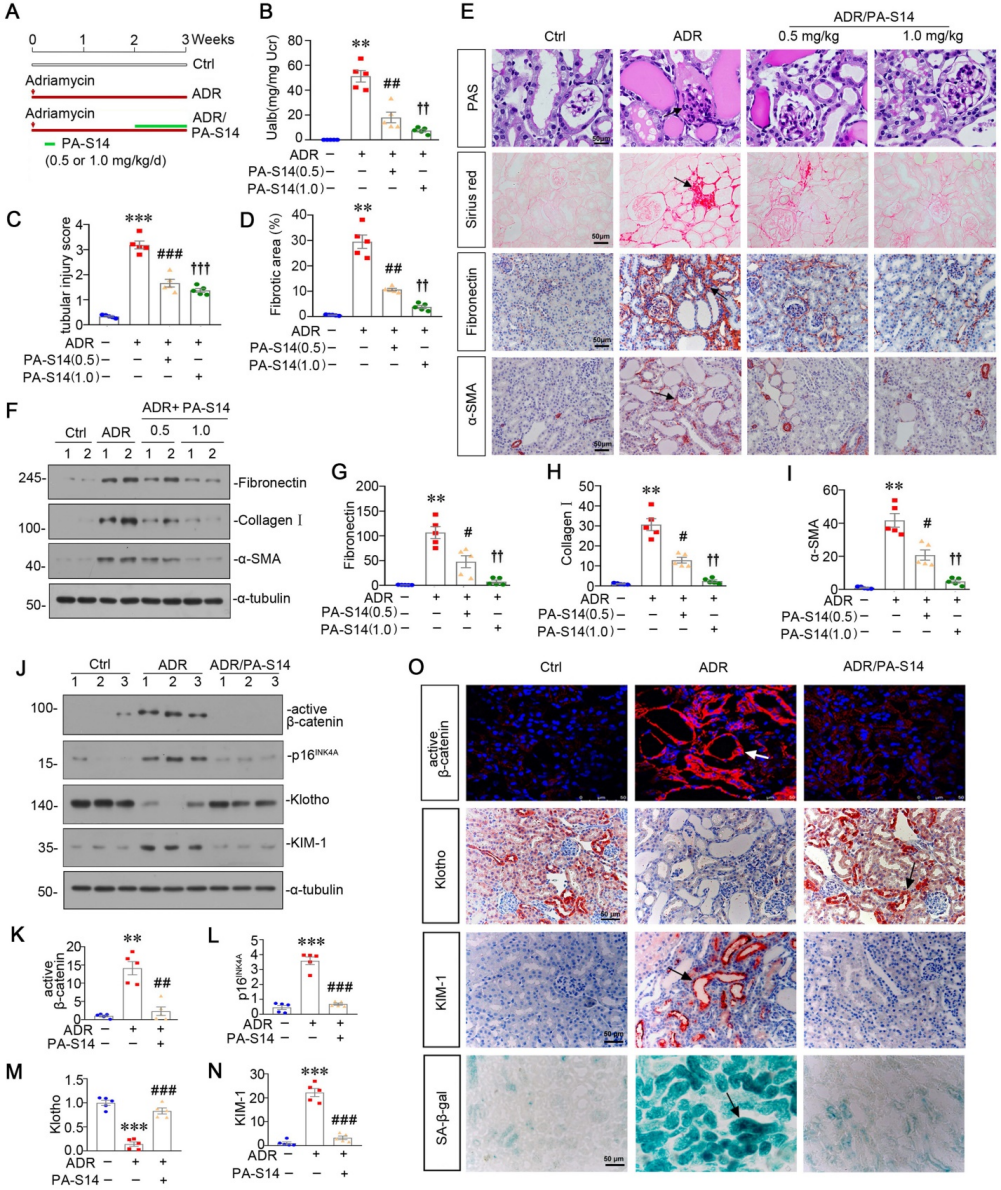
** PA-S14 ameliorates renal fibrosis and tubular cell senescence in adriamycin nephropathy.** (**A**) Diagram shows the experimental design. Red arrows indicate the time points for injection of adriamycin (ADR). Green line indicates PA-S14 treatment (0.5 mg/kg or 1.0 mg/kg body weight). (**B**) Urinary albumin levels in different groups at 3 weeks after ADR. Urinary albumin was expressed as milligrams per milligram creatinine. **P < 0.01 versus control mice; ##*P* < 0.01 versus ADR mice; ††*P* < 0.01 versus ADR mice (n = 5). (**C**) Quantitative analysis of tubular injury in different groups as indicated. Kidney sections were subjected to PAS staining. At least 10 randomly selected fields were evaluated under 400× magnification and results were averaged for each animal. ***P < 0.001 versus control mice; ###*P* < 0.001 versus ADR mice; †††*P* < 0.001 versus ADR mice (n = 5). (**D**) Graphical representations of the degree of kidney fibrotic lesions in different groups after quantitative determination of Sirius red staining intensity. **P < 0.01 versus control mice; ##P < 0.01 versus ADR mice; ††P < 0.01 versus ADR mice (n = 5). (**E**) Representative micrographs show tubular injury and Collagen deposition in different groups as indicated. Paraffin sections were subjected to Periodic acid-Schiff (PAS) staining and Sirius red staining, respectively. Representative micrographs showing renal expression of Fibronectin and α‐SMA in different groups. Arrows indicate positive staining. Scale bar, 50 µm. (**F-I**) Representative western blot (**F**) and quantitative data showing renal expression of Fibronectin (**G**), Collagen I (**H**), and α‐SMA (**I**) in different groups. Numbers (1-2) indicate each individual animal in a given group. **P < 0.01 versus control mice; #*P* < 0.05 versus ADR mice; ††*P* < 0.01 versus ADR mice (n = 5). (**J-N**) Representative western blot and quantitative data show renal expression of active-β-catenin, p16^INK4A^, Klotho and KIM-1 in different groups as indicated. Numbers (1-3) indicate each individual animal in given group. **P < 0.01, ***P < 0.001versus control mice; ##*P* < 0.01, ###*P* < 0.001 versus ADR mice (n = 5). (**O**) Representative staining micrographs show renal expression of active β-catenin, Klotho, KIM-1, and SA‐β‐gal activity. Frozen kidney sections were stained with an antibody against active β-catenin and assessed for SA‐β‐gal activity. Paraffin kidney sections were immunostained with the antibody against Klotho or KIM-1. Arrows indicate positive staining. Scale bar, 50 µm.

We then treated PA-S14 into mice at 2 weeks after adriamycin injection (Figure 7A), a time point when tubular cell injury and renal fibrosis are already established (Figure S6). We found in established renal fibrosis, PA-S14 also decreased urinary albumin secretion, inhibited tubular injury and retarded renal fibrosis (Figure 7B to D). The staining of PAS, Sirius red, fibronectin, and α-SMA also demonstrated PA-S14 effectively ameliorated tubular injury, and matrix deposition in interstitial area (Figure 7E). Similar results were observed when fibronectin, collagen I, and α-SMA were assessed by western blotting (Figure 7F to I).

We then assessed the effect of PA-S14 on tubular cell senescence. As shown (Figure 7J to N), PA-S14 strongly decreased the expression of active β-catenin, p16^INK4A^, and KIM-1, and preserved Klotho expression. Similar results were observed when active β-catenin, Klotho, and KIM-1 were assessed by immunostaining. Moreover, SA‐β‐gal activity staining also showed adriamycin-induced tubular senescence was largely inhibited by PA-S14 treatment (Figure 7O).

### PA-S14 promotes autophagy activity and mitochondrial homeostasis in ADR nephropathy through promoting LKB1/AMPK activation

**Figure 8:**
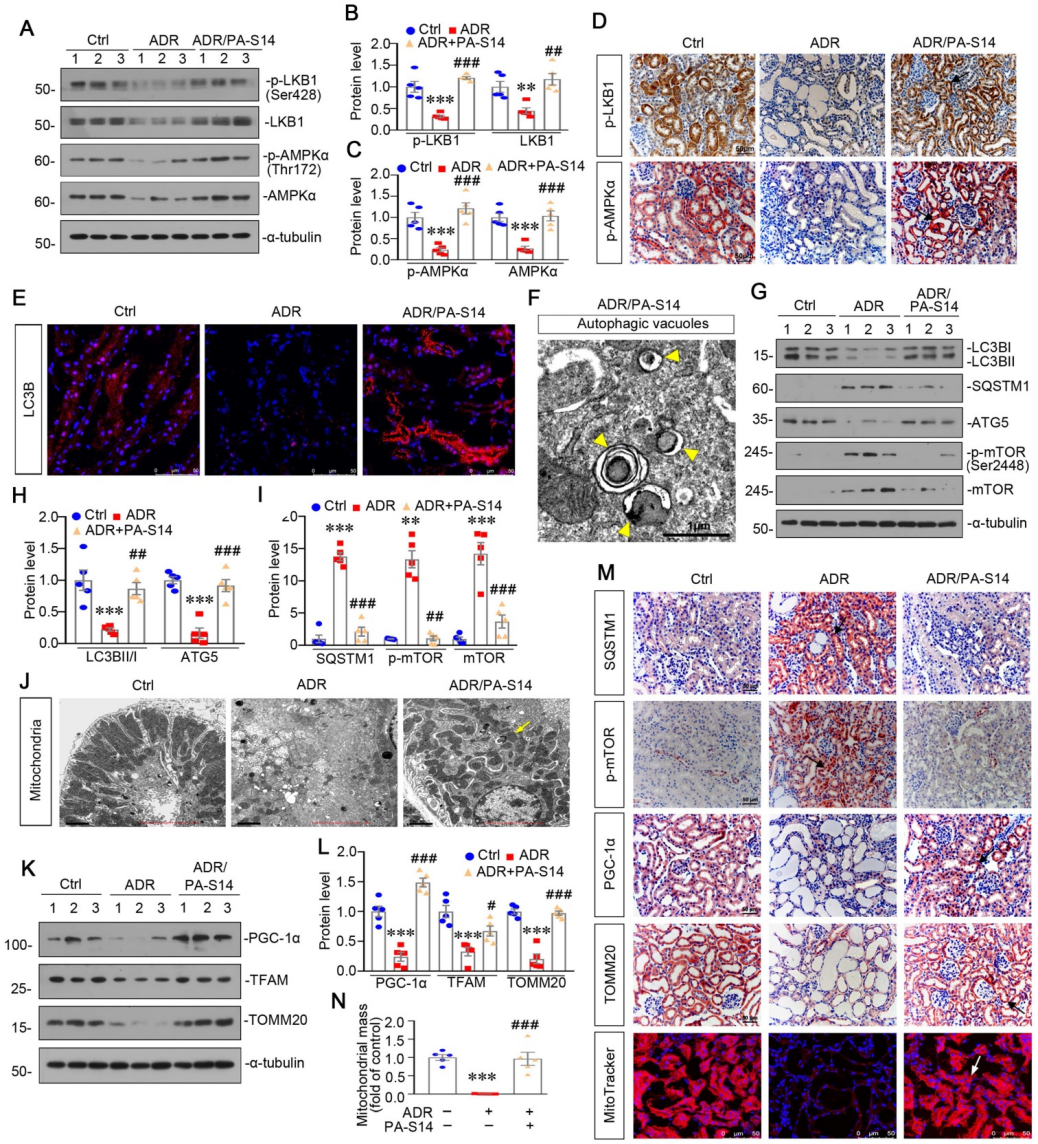
** PA-S14 induces autophagy activity and preserves mitochondrial homeostasis in ADR nephropathy through promoting LKB1/AMPK activation. (A-C)** Representative western blot and quantitative data showing renal expression of p-LKB1, LKB1, p-AMPKα, and AMPKα in different groups. Numbers (1-3) indicate each individual animal in a given group. **P < 0.01, ***P < 0.001 versus control mice; ##*P* < 0.01, ###*P* < 0.001 versus ADR mice (n = 5). **(D)** Representative micrographs show the p-LKB1 and p-AMPKα expression in different groups as indicated. Kidney sections were stained with antibodies against p-LKB1 and p-AMPKα, respectively. Arrows indicate positive staining. Scale bar, 50 µm. **(E)** Representative micrographs show the expression of LC3B. The frozen kidney sections were stained with an antibody against LC3B. Arrows indicate positive staining. Scale bar, 50 µm. **(F)** Ultrathin kidney sections were studied using a transmission electron microscope (TEM). TEM analyses show that PA-S14 increased autophagic vacuoles (yellow arrowheads). Scale bar, 1 µm. **(G-I)** Representative western blot and quantitative data showing renal expression of the LC3BII/I ratio, SQSTM1, ATG5, p-mTOR, and mTOR in different groups. Numbers (1-3) indicate each individual animal in a given group. **P < 0.01, ***P < 0.001 versus control mice; ##*P* < 0.01, ###*P* < 0.001 versus ADR mice (n = 5). **(J)** Ultrathin kidney sections were studied using a transmission electron microscope. Arrow indicates the normal round or rod‐shaped mitochondria and regular arrangement of mitochondrial cristae in the group treated with PA-S14. Scale bar, 1 µm. **(K-L)** Representative western blot (K) and quantitative data (L) show an increased expression of PGC-1α, TFAM and TOMM20 proteins in ADR kidneys after PA-S14 treatment. Numbers (1-3) indicate each individual animal in given group. ***P < 0.001 versus control mice; #*P* < 0.05, ###*P* < 0.001 versus ADR mice (n = 5). **(M)** Representative micrographs show the expression of SQSTM1, p-mTOR, PGC-1α, TOMM20. Paraffin kidney sections were used for immunohistochemistry staining. The frozen kidney sections were stained with MitoTracker deep red (3 µm) probe. DAPI (4′,6‐diamidino‐2‐phenylindole) was used to stain the nuclei (blue). Arrows indicate positive staining. Scale bar, 50 µm. **(N)** Quantification of renal mitochondrial mass is shown. Mitochondrial mass was determined by the fluorescence intensity of MitoTracker deep red staining normalized to DAPI. ***P < 0.001 versus control mice; ###*P* < 0.001 versus ADR mice (n = 5).

We then analyzed LKB1/AMPK signaling. As shown (Figure 8A to D), the expression of p-LKB1, LKB1, p-AMPKα and AMPKα, was decreased by ADR injection, but significantly reversed by PA-S14 treatment. qRT-PCR analyses indicated that treatment with PA-S14 enhanced the expression of LKB1 mRNA in ADR mice (Figure S7). We then analyzed the effects of PA-S14 on autophagy. As shown, PA-S14 induced LC3B dot accumulation, and increased autophagic vacuoles (Figure 8E and F). Western blotting analysis of LC3B, SQSTM1, ATG5, p-mTOR, and mTOR also revealed PA-S14 triggered autophagy activity (Figure 8G to I, and M). We next assessed mitochondrial biogenesis. As shown, PA-S14 perfectly preserved the mitochondrial structure and numbers (Figure 8J), and restored the expression of PGC-1α and TFAM, the two master regulators governing mitochondrial biogenesis, and TOMM20 27, a marker for mitochondrial mass (Figure 8K and L). Similar results were observed when PGC-1α and TOMM20 were tested by immunostaining (Figure 8M). MitoTracker staining also confirmed PA-S14 greatly restored mitochondrial mass in ADR mice (Figure 8M and N).

### PA-S14 ameliorates renal fibrosis and tubular cell senescence in 5/6NX mice

**Figure 9:**
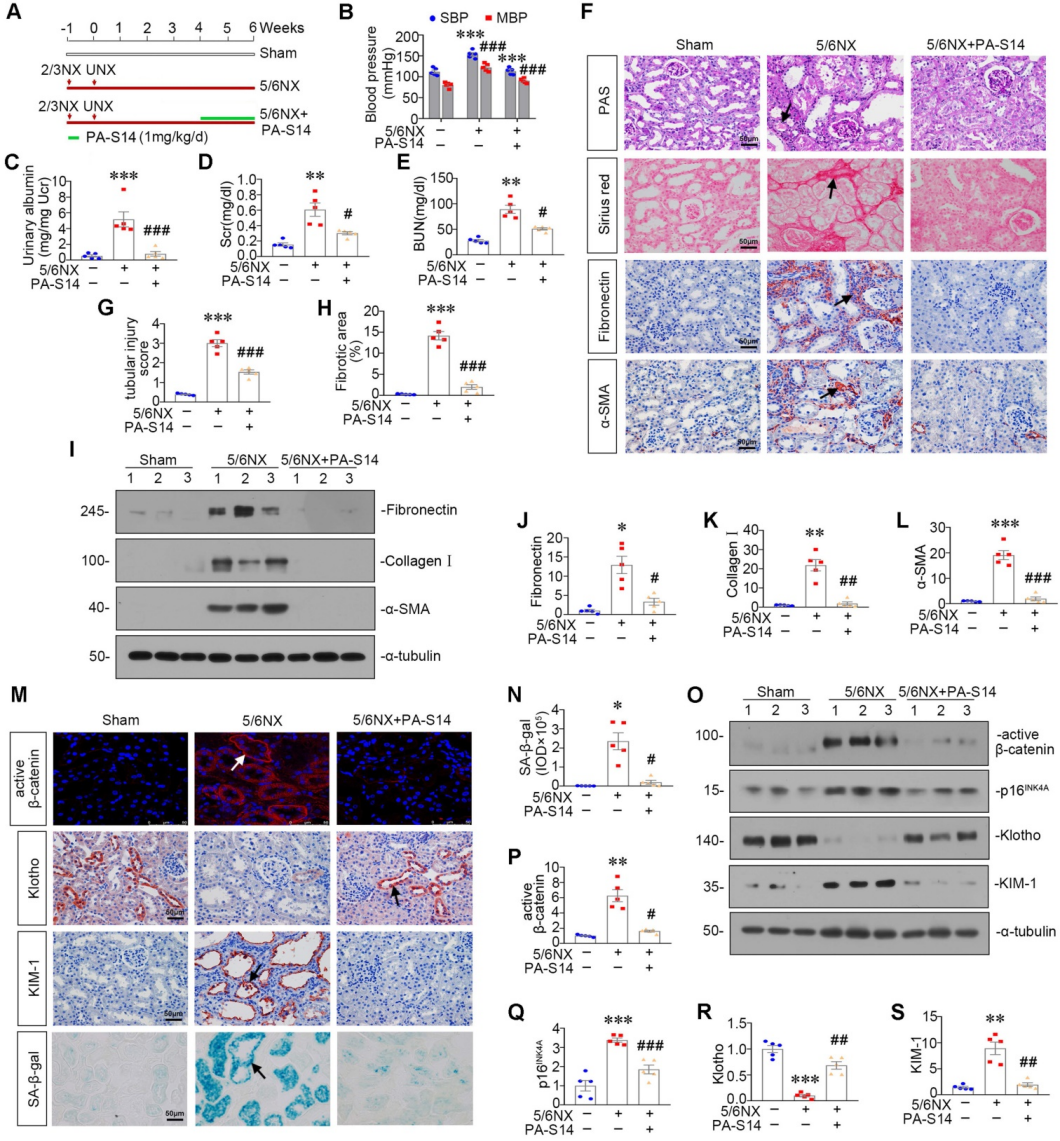
** PA-S14 attenuates renal fibrosis and tubular cell senescence in 5/6NX mice. (A)** Diagram shows the experimental design. Red arrows indicate the surgical resection of left kidney and nephrectomy of right kidney. Green line indicates PA-S14 treatment (1.0 mg/kg body weight). **(B)** Tail-cuff blood pressure measurements show that PA-S14 normalizes systolic blood pressure (SBP) and mean blood pressure (MBP) in mice at 6 weeks after 5/6NX. **(C)** Urinary albumin levels in different groups at 6 weeks after 5/6NX. ***P < 0.001 versus sham mice; ###*P* < 0.001 versus 5/6NX mice (n = 5). **(D-E)** The treatment of PA-S14 significantly decreased serum creatinine and BUN levels. **P < 0.01 versus sham mice; #*P* < 0.05 versus 5/6NX mice (n = 5). **(F)** Representative micrographs show tubular injury and Collagen deposition in different groups as indicated. Paraffin sections were subjected to PAS staining and Sirius red staining, respectively. Representative micrographs showing renal expression of Fibronectin and α‐SMA in different groups. Arrows indicate positive staining. Scale bar, 50 µm. **(G)** Quantitative analysis of tubular injury in different groups as indicated. Kidney sections were subjected to PAS staining. At least 10 randomly selected fields were evaluated under 400× magnification and results were averaged for each animal. ***P < 0.001 versus sham mice; ###*P* < 0.001 versus 5/6NX mice (n = 5). **(H)** Graphical representations of the degree of kidney fibrotic lesions in different groups after quantitative determination of Sirius red staining intensity. ***P < 0.001 versus sham mice; ###*P* < 0.001 versus 5/6NX mice (n = 5). **(I-L)** Representative western blot and quantitative data show the reduction of renal Fibronectin, Collagen I and α‐SMA expression after PA-S14 treatment. Numbers (1-3) indicate each individual animal in given group. *P < 0.05, **P < 0.01, ***P < 0.001 versus sham mice; #*P* < 0.05, ##*P* < 0.01, ###*P* < 0.001 versus 5/6NX mice (n = 5). **(M)** Representative micrographs show the expression of active β-catenin, SA‐β‐gal activity, Klotho and KIM-1 in different groups as indicated. Frozen kidney sections were stained for active β-catenin and SA‐β‐gal activity. Paraffin sections were stained with antibodies against Klotho and KIM-1, respectively. Arrows indicate positive staining. Scale bar, 50 µm. **(N)** Quantitative determination of SA‐β‐gal activity staining in 5/6NX kidney. *P < 0.05 versus sham mice; #*P* < 0.05 versus 5/6NX mice (n = 5). **(O-S)** Representative western blot and quantitative data showing renal expression of active β-catenin, p16^INK4A^, Klotho and KIM-1. Numbers (1-3) indicate each individual animal in a given group. **P < 0.01, ***P < 0.001 versus sham mice; #*P* < 0.05, ##*P* < 0.01, ###*P* < 0.001 versus 5/6NX mice (n = 5).

We further observed the effects of PA-S14 in 5/6NX mice, a chronic renal failure model (Figure 9A). As shown, both systolic blood pressure (SBP) and mean blood pressure (MBP) increased in 5/6NX mice, however, treatment with PA-S14 significantly inhibited the increase in SBP and MBP (Figure 9B). We then evaluated the protective effects of PA-S14 on renal function. As shown in Figure 9C to E, PA-S14 significantly decreased the secretion of urinary albumin, and reduced the serum creatinine (Scr) and blood urea nitrogen (BUN) levels.

We further analyzed renal tubular cell injury and fibrosis. PAS and Sirius red staining (Figure 9F to H) showed renal tubular injury and interstitial fibrosis were greatly inhibited by PA-S14. PA-S14 also greatly decreased the expression of fibronectin and α‐SMA. The similar results were observed when fibronectin, collagen I, and α‐SMA were examined by western blotting (Figure 9I to L). We then evaluated cellular senescence. As shown in Figure 9M and N, PA-S14 strongly inhibited the expression of active β-catenin, KIM-1 and SA‐β‐gal activity, but restored the expression of Klotho. Similar results were observed by western blotting (Figure 9O to S). We also observed treatment with PA-S14 greatly decreased the expression of p16^INK4A^, a maker of cellular senescence (Figure 9O and Q).

### PA-S14 induces autophagy and restores mitochondrial homeostasis in 5/6NX mice via activating LKB1/AMPK signaling

**Figure 10:**
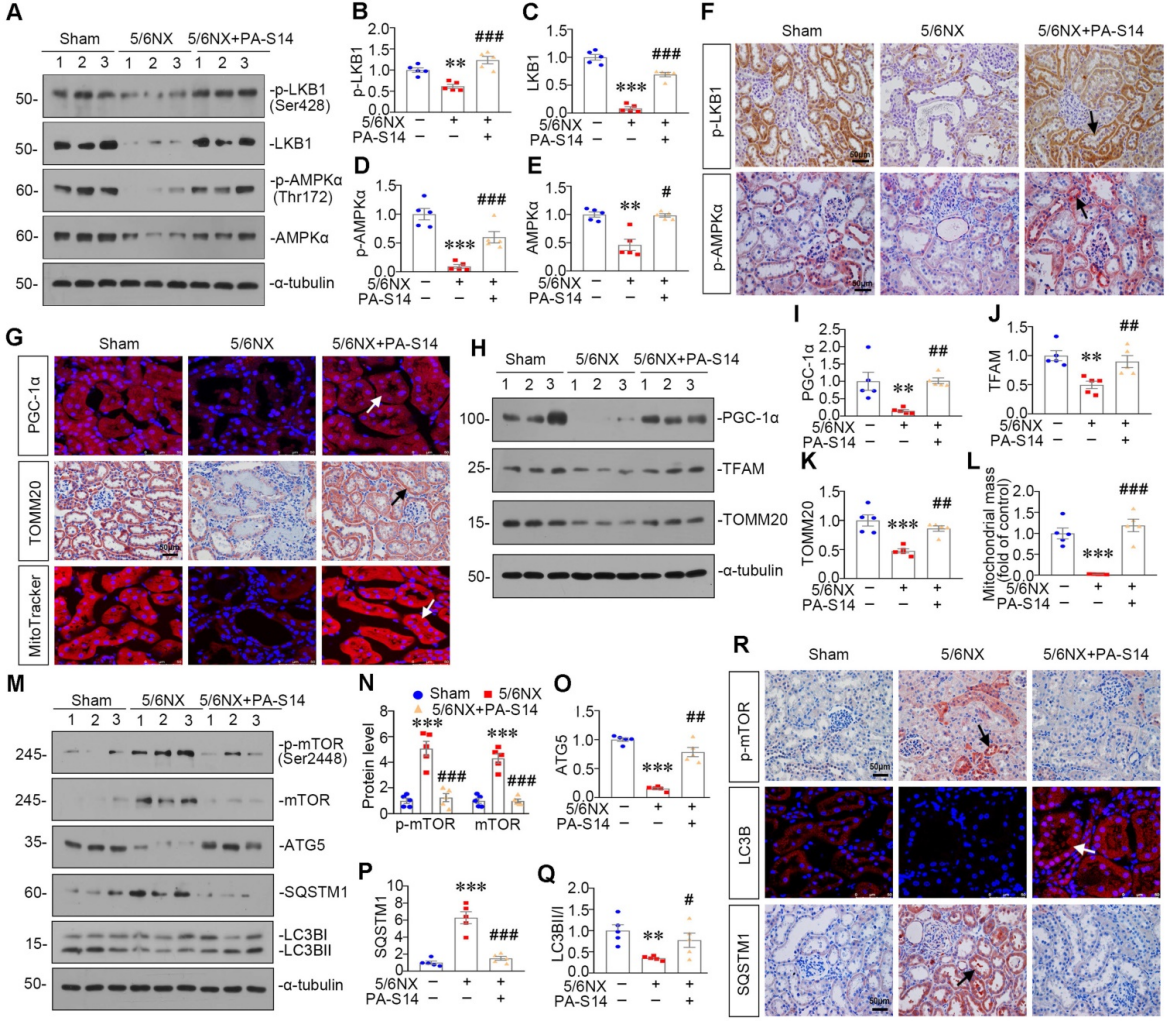
** PA-S14 induces autophagy and restores mitochondrial biogenesis in 5/6 NX mice via activating LKB1/AMPK signaling. (A-E)** Representative western blot and quantitative data showing renal expression of p-LKB1, LKB1, p-AMPKα, and AMPKα in different groups. Numbers (1-3) indicate each individual animal in a given group. **P < 0.01, ***P < 0.001 versus sham mice; #*P* < 0.05, ###*P* < 0.001 versus 5/6NX mice (n = 5). **(F)** Representative images show the p-LKB1 and p-AMPKα staining in different groups as indicated. Arrows indicate positive staining. Scale bar, 50 µm. **(G)** PGC-1α staining and TOMM20 staining of Paraffin kidney sections from 5/6 NX mice treated with or without PA-S14. The frozen kidney sections were stained with MitoTracker deep red (3 µm) probe. DAPI (4′,6‐diamidino‐2‐phenylindole) was used to stain the nuclei (blue). Arrows indicate positive staining. Scale bar, 50 µm. **(H-K)** Representative western blot and quantitative data showing renal expression of the PGC‐1α, TFAM and TOMM20 in different groups. Numbers (1-3) indicate each individual animal in a given group. **P < 0.01, ***P < 0.001 versus sham mice; ##*P* < 0.01 versus 5/6NX mice (n = 5). **(L)** Quantification of renal mitochondrial mass is shown. Mitochondrial mass was determined by the fluorescence intensity of MitoTracker deep red staining normalized to DAPI. ***P < 0.001 versus sham mice; ###*P* < 0.001 versus 5/6NX mice (n = 5). **(M-Q)** Representative western blot and quantitation of the renal expressions of the LC3BII/I ratio, SQSTM1, ATG5, p-mTOR, and mTOR in different groups. Numbers (1-3) indicate each individual animal in a given group. **P < 0.01, ***P < 0.001 versus sham mice; #*P* < 0.05, ##*P* < 0.01, ###*P* < 0.001 versus 5/6NX mice (n = 5). **(R)** Representative micrographs show renal expression of p-mTOR, LC3B, and SQSTM1. Paraffin kidney sections were stained with antibodies against p-mTOR and SQSTM1. The frozen kidney sections were stained with an antibody against LC3B. Arrows indicate positive staining. Scale bar, 50 µm.

We then examined LKB1/AMPK signaling. As shown, pLKB1 and LKB1, and p-AMPKα and AMPKα, were greatly restored by PA-S14 in 5/6NX mice (Figure 10A to F). The mRNA expression of LKB1 was also increased in PA-S14-treated mice (Figure S7). We further examined mitochondrial homeostasis. As shown (Figure 10G to J), the expressional levels of PGC-1α and TOMM20 were reduced in 5/6NX mice, however, they were strongly induced by PA-S14 treatment. MitoTracker staining also showed mitochondria mass was preserved after treatment with PA-S14 (Figure 10G and L). We also assessed the autophagic pathway. As shown, PA-S14 blocked the induction of p-mTOR, mTOR and SQSTM1 in 5/6NX mice, but restored the expression ATG5 and the conversion of LC3BI to II (Figure 10M to Q). Furthermore, immunostaining of p-mTOR, LC3B and SQSTM1 also revealed PA-S14 evidently promoted autophagy pathway (Figure 10R).

### PA-S14 attenuates renal fibrosis and tubular cell senescence in UUO mice

**Figure 11:**
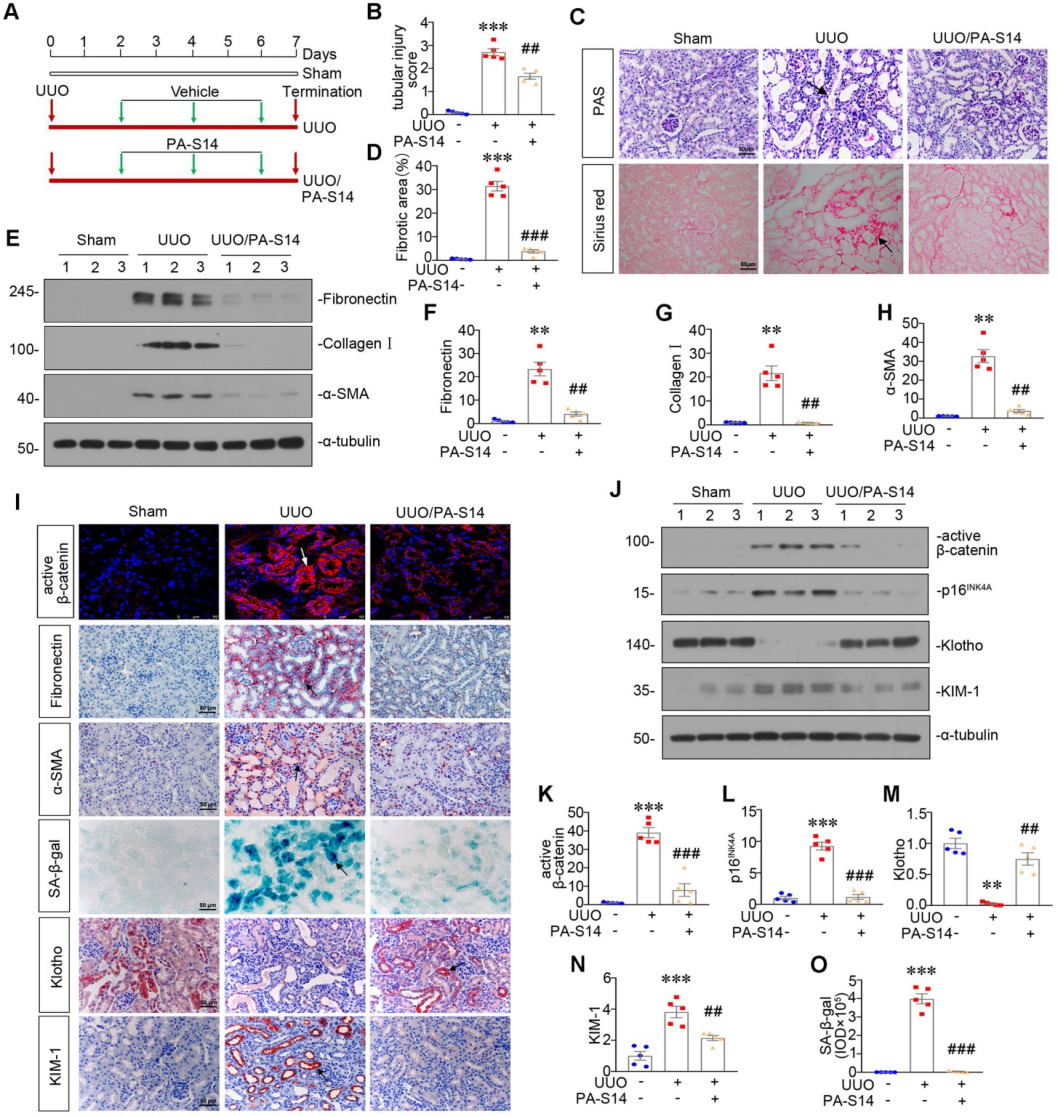
** PA-S14 attenuates renal fibrosis and tubular cell senescence in UUO mice. (A)** Diagram shows the experimental design. Red arrows indicate the time points undergoing UUO and sacrifice, respectively. Green arrows indicate PA-S14 treatment (1.0 mg/kg body weight). **(B)** Quantitative analysis of tubular injury in different groups as indicated. Kidney sections were subjected to PAS staining. At least 10 randomly selected fields were evaluated under 400× magnification and results were averaged for each animal. ***P < 0.001 versus sham controls; ##*P* < 0.01versus UUO (n = 5). **(C)** Representative staining micrographs show Periodic acid-Schiff (PAS) and Sirius red staining. Paraffin sections were performed to PAS and Sirius red staining. Arrows indicate positive staining. Scale bar, 50 µm. **(D)** Graphical representations of the degree of kidney fibrotic lesions in different groups after quantitative determination of Sirius red staining intensity. ***P < 0.001 versus sham controls; ###*P* < 0.001 versus UUO (n = 5). **(E-H)** Representative western blot and quantitative data show the reduction of renal Fibronectin, Collagen I and α‐SMA expression after PA-S14 treatment. Numbers (1-3) indicate each individual animal in given group. **P < 0.01 versus sham controls; ##*P* < 0.01 versus UUO (n = 5). (I) Representative micrographs show the expression of active β-catenin, Fibronectin, α‐SMA, SA‐β‐gal activity, Klotho and KIM-1 in different groups as indicated. Frozen kidney sections were stained for active β-catenin and SA‐β‐gal activity. Paraffin sections were stained with antibodies against Fibronectin, α‐SMA, Klotho, and KIM-1, respectively. Arrows indicate positive staining. Scale bar, 50 µm. **(J-N)** Representative western blot and quantitative data showing renal expression of active β-catenin, p16^INK4A^, Klotho and KIM-1. Numbers (1-3) indicate each individual animal in a given group. **P < 0.01, ***P < 0.001 versus sham controls; ##*P* < 0.01, ###*P* < 0.001versus UUO (n = 5). **(O)** Quantitative determination of SA‐β‐gal activity staining in UUO kidney. ***P < 0.001 versus sham controls; ###*P* < 0.001 versus UUO (n = 5).

We also examined the protective role of PA-S14 in UUO mice. The detailed experimental design is shown in Figure 11A. PA-S14 was administered by intermittent treatment. We first analyzed tubular cell injury and fibrotic lesions. As shown in Figure 11B to H, PA-S14 significantly ameliorated renal tubular injury and fibrotic lesions, and reduced the expression of fibronectin, collagen I, and α‐SMA. Similar results were observed when fibronectin and α‐SMA were tested by immunostaining (Figure 11I). We next assessed cellular senescence. As shown in Figure 11I, the expression of active β-catenin and SA‐β‐gal activity were increased in UUO mice, but greatly decreased by PA-S14 treatment. Furthermore, PA-S14 restored the expression of Klotho and decreased KIM-1. Similar results were observed when active β-catenin, p16^INK4A^, Klotho, and KIM-1 were assessed by western blotting (Figure 11J to O).

### PA-S14 induces autophagy and restores mitochondrial homeostasis in UUO mice via activating LKB1/AMPK signaling

**Figure 12:**
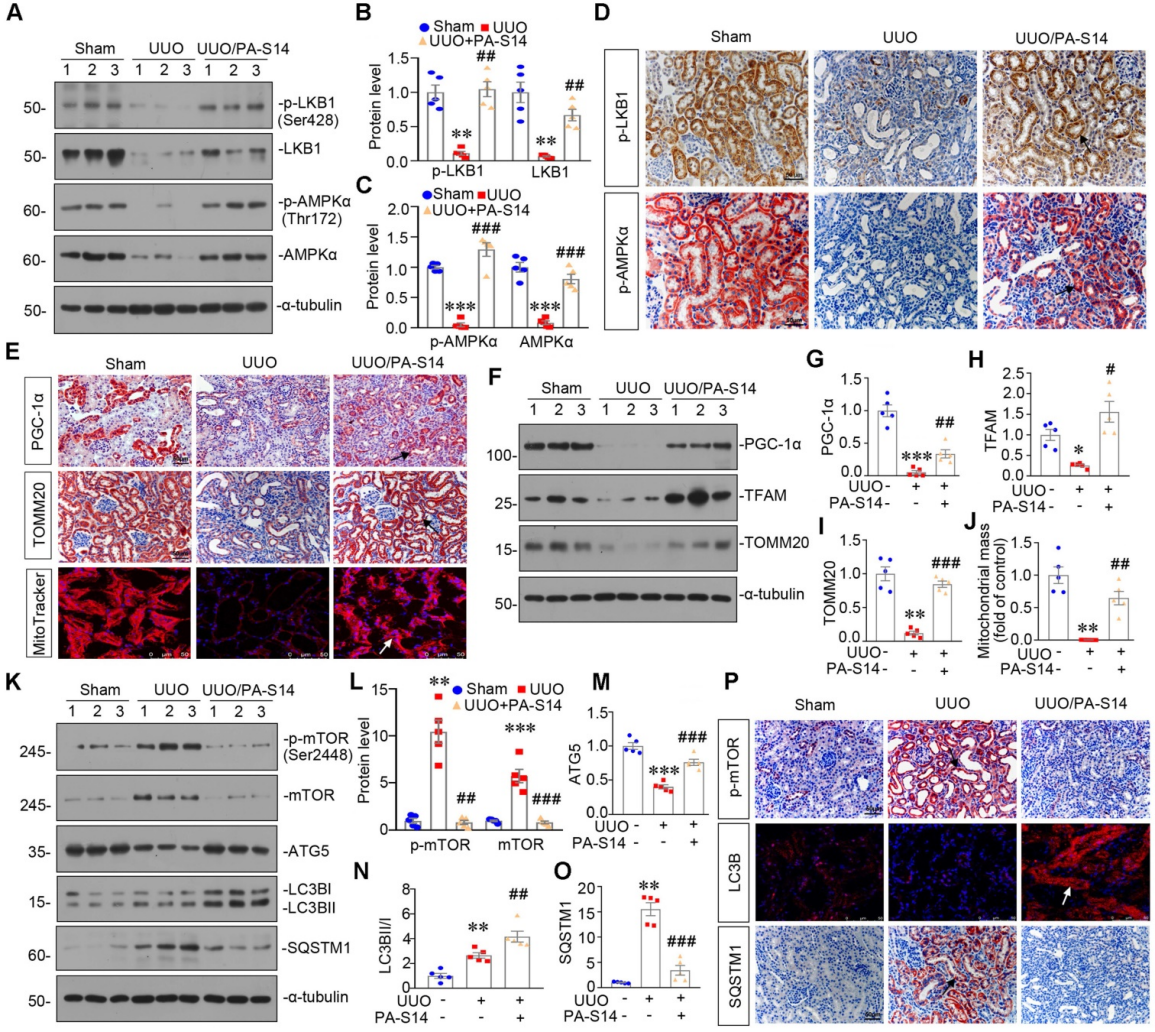
** PA-S14 induces autophagy and restores mitochondrial biogenesis in UUO mice via activating LKB1/AMPK signaling. (A-C)** Representative western blot and quantitative data showing renal expression of p-LKB1, LKB1, p-AMPKα, and AMPKα in different groups. Numbers (1-3) indicate each individual animal in a given group. **P < 0.01, ***P < 0.001 versus sham controls; ##*P* < 0.01, ###*P* < 0.001 versus UUO (n = 5). **(D)** Representative micrographs show the p-LKB1 and p-AMPKα expression in different groups as indicated. Kidney sections were stained with antibodies against p-LKB1 and p-AMPKα. Arrows indicate positive staining. Scale bar, 50 µm.** (E)** Representative micrographs show the expression of PGC-1α, TOMM20 and Mitochondrial mass. Paraffin kidney sections were stained with antibodies against PGC-1α and TOMM20. The frozen kidney sections were stained with MitoTracker deep red (3 µm) probe. DAPI (4′,6‐diamidino‐2‐phenylindole) was used to stain the nuclei (blue). Arrows indicate positive staining. Scale bar, 50 µm. **(F-I)** Representative western blot and quantitative data showing renal expression of the PGC‐1α, TFAM and TOMM20 in different groups. Numbers (1-3) indicate each individual animal in a given group. *P < 0.05, **P < 0.01, ***P < 0.001 versus sham controls; #*P* < 0.05, ##*P* < 0.01, ###*P* < 0.001 versus UUO (n = 5). **(J)** Quantification of renal mitochondrial mass is shown. Mitochondrial mass was determined by the fluorescence intensity of MitoTracker deep red staining normalized to DAPI. **P < 0.01 versus sham controls; ##*P* < 0.01 versus UUO (n = 5). **(K-O)** Representative western blot and quantitative data showing renal expression of the LC3BII/I ratio, SQSTM1, ATG5, p-mTOR, and mTOR in different groups. Numbers (1-3) indicate each individual animal in a given group. **P < 0.01, ***P < 0.001 versus sham controls; ##*P* < 0.01, ###*P* < 0.001 versus UUO (n = 5). **(P)** Representative micrographs show renal expression of p-mTOR, LC3B, and SQSTM1. Paraffin kidney sections were stained with antibodies against p-mTOR and SQSTM1. The frozen kidney sections were stained with an antibody against LC3B. Arrows indicate positive staining. Scale bar, 50 µm.

LKB1/AMPK pathway was also examined. As shown in Figure 12, A to D and S7, p-LKB1 and LKB1, p-AMPKα and AMPKα, LKB1 mRNA were greatly restored by PA-S14 in UUO mice. We next assessed mitochondrial homeostasis. As shown in Figure 12E to J, the expressional levels of PGC-1α, TOMM20, and TFAM, were deceased in UUO mice, however, they were strongly resored by PA-S14 treatment. Mitochondrial mass was examined by MitoTracker staining. As shown, it was reduced in UUO mice, but strongly enhanced after treatment with PA-S14 (Figure 12E and J). We next assessed the autophagic pathway. As shown, PA-S14 significantly decreased the expression of p-mTOR, mTOR and SQSTM1, and upregulated the expression of ATG5 and the conversion of LC3BI to II (Figure 12K to O). Furthermore, the immunostaining of p-mTOR, LC3B and SQSTM1 also revealed PA-S14 evidently promoted autophagy (Figure 12P).

We also observed whether PA-S14 took effects on MARK2, SIK, and PP2C-α, the other AMPK-related kinases, and found it was not (Figure S8). In addition, we also examined whether PA-S14 induced LKB1 signals in other cell types such as podocytes, mesangial cells, and renal fibroblasts, and also found it was not (Figure S8). All these results suggest PA-S14 specifically induces LKB1 signaling in renal tubular epithelial cells.

**Figure 13:**
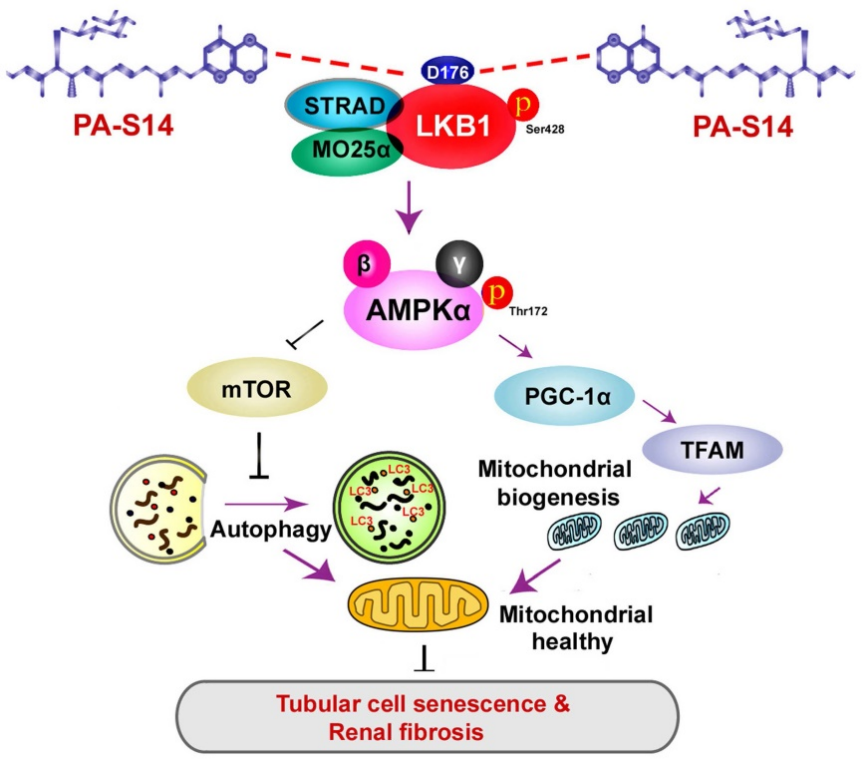
The schematic presentation depicts the potential mechanism by which PA-S14 inhibits tubular cell senescence and renal fibrosis. As an activator of LKB1, PA-S14 may attenuate tubular cell senescence and renal fibrosis by promoting autophagy and mitochondrial biogenesis. The underlying mechanisms are related to its activation of AMPKα. These effects lead to mitochondrial healthy, which further reduces tubular cell senescence and retards renal fibrosis.

Hence, as summarized in Figure 13, we concluded PA-S14 is a new activator of LKB1. Through binding with D176 in the kinase domain of LKB1, PA-S14 further triggers AMPK-induced autophagy and mitochondrial biogenesis. This leads to mitochondrial homeostasis, which collectively protects against tubular cell senescence and renal fibrosis.

## Discussion

Renal tubular cell is the principle cell type in kidney parenchyma 28. The energy metabolism malfunction in tubular cells is highly related with renal fibrosis 7, 29. However, the therapeutic strategies are still underdetermined.

Mitochondrion is one of the most important organelles in eukaryotic cells. There are abundant mitochondria in renal tubular epithelial cells to meet the high need of energy 30. Energy metabolism disturbance is the common feature in tubular cell injury, which is critical for the decline of renal function 31. A better quality control of mitochondria is crucial for maintaining cell homeostasis. Otherwise, renal tubular cells would go into apoptosis or senescence 32. Mitochondrial biogenesis is controlled by PGC-1α, which promotes OXPHOS gene transcription and translation through inducing TFAM, the mitochondrial transcription factor 33. On the other side, damaged mitochondria could be degraded by autophagy, a highly conserved self-defense process.

Through degrading and recycling wastes, autophagy plays a key role in maintaining cell homeostasis and regulating organ function 34, 35. Autophagy deficiency plays a crucial role in the damage in renal tubular cells 36. With the vigorous metabolism, renal tubular cells would produce a considerable amount of wastes which needs to be cleared and recycled, especially damaged mitochondria 37. Of note, autophagy could selectively degrade damaged mitochondria, i.e. mitophagy 31, playing a key role in mitochondrial homeostasis. However, autophagy is defective in kidney diseases. Moreover, the therapeutic drugs to enhance autophagy are still very limited.

LKB1 is a serine/threonine kinase, and is a critical metabolic checkpoint in controlling energy storage and cellular homeostasis. LKB1 deletion in tubules decreased energy supply and induced progressive kidney disease 22. LKB1 mediates AMPK activity through activating the phosphorylation of 172 threonine site on the AMPK α subunit 38. Furthermore, AMPK activates PGC-1α 39 to control mitochondrial biogenesis 40, 41, and induces autophagy through inhibiting mammalian target of rapamycin complex (mTORC)1 signaling and triggering Beclin1 and ATG5 activity 42, 43. Hence, LKB1/AMPK signaling plays a key role in energy homeostasis through the regulation of both mitochondrial homeostasis and autophagy 44. However, LKB1 activators are not commercially available yet.

Human LKB1 protein consists of a highly conserved kinase domain with residues 50 to 319 18-20. Several residue mutations, such as L67, K78, D176, R304 and W308, have been identified to be involved in loss of the function in LKB1 protein 21. These indicate these sites could be explored for rational drug design. PA-S14 is a new molecule isolated from the strain Streptomyces psammoticus SCSIO NS126 in a mangrove sediment sample by our group. In this study, we found PA-S14 acts as a novel direct LKB1 activator through binding with residue D176 in kinase domain.

PA-S14 is a glycosylated and hydroxylative derivative of piericidin A (PA), an inhibitor to respiratory complex I. Compared with PA, PA-S14 exhibited a low toxicity and a quick and long-lasting activation on complex I activity and ATP production (Figure 2). Of interest, we found PA-S14 persistently induces a high-level activation effect on LKB1 and AMPK, which is not observed in PA (Figure S3). We further found PA-S14 is a potent activator of LKB1. Consequently, PA-S14 activates AMPK to induce autophagy and mitochondrial biogenesis. Furthermore, PA-S14 effectively protected against renal tubular cell senescence and renal fibrosis.

There are several lines of evidences to support our findings. First, PA-S14 quickly induced LKB1/AMPK signaling, with no inhibitory effects on ATP production and complex I activity. This suggests the direct stimulatory effects of PA-S14 on LKB1. Indeed, we found PA-S14 binds with LKB1 kinase domain, especially the residue D176. PA-S14 induced LKB1 phosphorylation and activation, and the formation of LKB1, MO25 and STRAD complex, suggesting its potent effect on LKB1 activation (Figures 2 and 3). Second, even in healthy mice, PA-S14 induced LKB1/AMPK signaling, and triggered autophagy and mitochondrial biogenesis (Figure 4). Third, in diseased models such as UIRI, ADR, UUO, and 5/6NX, PA-S14 treatment exhibited superior therapeutic effects against cellular senescence and fibrogenesis through inducing autophagy and mitochondrial biogenesis pathways (Figures 5 to 12). All these results clearly demonstrated PA-S14 is a novel LKB1 activator.

Most drugs pass through the cell membrane through simple diffusion depending on concentration differences, also called a passive transmission mode. PA-S14 contains a hydrophobic methylated polyketide side chain, which could be possibly dissolved in the lipid layer of the cell membrane and help to pass through it. Therefore, we thought PA-S14 gets into a cell through the passive transmission mode. Certainty, this should be investigated in detail in the future.

LKB1 could modulate a variety of cellular functions ranging from energy metabolism, cellular senescence to malignancy. LKB1 is highly expressed in normal kidney, but loss of function in diseased state. Renal tubular cell has vigorous metabolism. Hence, LKB1 plays a key role in its survival and energy homeostasis. However, the direct LKB1 activators are not available in commercial.

Fortunately, we found PA-S14 acts as a novel lead compound of LKB1 activator to effectively protect against renal fibrosis. It is a natural piericidin glycoside analogue obtained from the culture broth of a marine-derived *Streptomyces* strain. We further identified the residue D176 in LKB1 kinase domain is the key binding site of PA-S14. As one of the autophosphorylation sites, D176 mutation has been proved to relate with the loss of LKB1. Although more studies are needed, our study strongly suggests PA-S14 is a potent potential of therapeutic strategies in CKD. We found PA-S14 binds with D176. This suggests PA-S14 may have a broad range of application beyond kidney area.

## Materials and Methods

Detailed Supplementary Methods are available at website.

### PA-S14 preparation

The strain *Streptomyces psammoticus* SCSIO NS126 was isolated from a mangrove sediment sample collected from the Pearl River estuary to South China Sea 45. The culture broth was extracted with ethyl acetate, and then concentrated. The extract was chromatographed to give eight fractions (Frs.1~8). Frs.2 was purified by silica gel to obtain a pure compound, which was identified as piericidin A (PA) as reported 45. Using HPLC analysis, Frs.7 was purified again to give six sub-fractions (Frs.7-1~7-6). Frs.7-4 was purified by semipreparative HPLC to obtain a piericidin analogue compound labeled as S14 (PA-S14). PA-S14 was indicated as a hydroxylative product of glucopiericidin A for its molecular weight 593 by MS analysis (Figure S2). It was determined to be 13-hydroxyglucopiericidin A by comparison of its ^1^H and ^13^C NMR date (Figure S2) with literature 46. The obtained compounds, PA-S14, determined to have ≥95% purity by analytical HPLC, were stored at -20 °C until use and dissolved in DMSO to a stock concentration of 10 mM.

### Animal models

Male BALB/c or CD-1 mice (20-25g) were obtained from Southern Medical University Animal Center, and were constructed for ADR, UUO, UIRI and 5/6NX models 47, 48.

For ADR model, BALB/c mice were administered a single intravenous injection of ADR (doxorubicin hydrochloride; Sigma, St. Louis, MO) at 11 mg/kg body weight. For UUO model, BALB/c mice were carried out by double-ligating the left ureter following a midline abdominal incision. For UIRI model, BALB/c mice were subjected to unilateral ischemia-reperfusion (IRI) surgery by an established protocol as described previously 47. For the 5/6NX model, male CD-1 mice were removed off the upper and lower poles of the left kidney, two thirds of the left kidney, and the right kidney one week later. The detailed experimental designs were shown in Figures 5A, 7A, 9A and 11A. Mice were intraperitoneally injected with PA-S14 at 0.5 or 1.0 mg/kg/d for indicated time. Blood pressure was measured by non-invasive tail cuff method as previously described 49. All animal studies were performed adhering to the Health Guide for the Care and Use of Laboratory Animals and approved by the Experimental Animal Committee at the Nanfang Hospital, Southern Medical University (NFYY-2017-0425).

### Urinary albumin, serum creatinine and BUN assay

Urinary albumin was measured using a commercial kit (Bethyl Laboratories, Montgomery, TX). Serum creatinine and BUN levels were determined by an automatic chemistry analyzer (AU480 Chemistry Analyzer, Beckman Coulter, Atlanta, Georgia).

### Cell culture and treatment

Human proximal tubular epithelial cells (HKC-8) were provided by Dr. Lorraine C. Racusen (Johns Hopkins University, Baltimore, MD), and cultured as described previously 50. LKB1 siRNA (siLKB1) sequence and negative control (siNC) were described as previously 51. HKC-8 cells were transfected with adenovirus expressing LKB1 shRNA (sh-LKB1) (HanBio Technology, Shanghai, China). Hela cells were also cultured in MEM supplemented with 10% fetal calf serum. Cell viability was assessed by the MTT (3-(4,5-dimethylthiazol-2-yl)-2,5-diphenyl-tetrazolium bromide) 52.

### Detection of Autophagic Flux

#### LC3 Puncta accumulation

HKC-8 cells were transfected with pCMV-GFP-LC3B plasmid (Beyotime Biotechnology**,** D2815, Beijing, China). GFP-LC3B aggregates on the autophagosome membrane in the form of spots (LC3B dots or punctae).

### mRFP-GFP-LC3B lentivirus infection

HKC-8 cells were infected with lentivirus expressing a tandem RFP-GFP-LC3B fusion protein (Hanbio Biotechnology, HB-LP210). Autophagosomes exhibit yellow dots (RFP and GFP signal), whereas autophagolysosomes exhibit red dots (RFP signal).

### Western blot analysis

Protein samples were prepared and electrotransfered onto PVDF membrane. PVDF membrane was blocked in 5% of milk, and incubated with different primary antibodies. The primary antibodies used were shown in supplementarty data. The next day, PVDF membrane was incubated with a responding secondary antibody and observed by chemiluminescent detection 50.

### qRT-PCR

Total RNA was obtained using a TRIzol RNA isolation system (Life Technologies, Grand Island, NY). Real-time PCR was performed on an ABI PRISM 7000 Sequence Detection System (Applied Biosystems, Foster City, CA). The sequences of the primer pairs used in qRT-PCR were shown in supplementary data.

### Histology, immunohistochemical and immunofluorescence staining

Paraffin-embedded kidney sections were performed with periodic acid-Schiff (PAS) and Sirius red staining to identify injured tubules and collagen deposition 53. Immunohistochemical and immunofluorescence staining were performed as described previously 50.

### SA‐β‐gal, mitoSOX, and MitoTracker staining

Frozen sections (3 μm) or cultured cells were performed β‐galactosidase activity staining (Cell Signaling Technology, 9860S), MitoTracker deep red (Thermo Fisher, M22426), and mitoSOX (Thermo Fisher, M36008) according to the manufacturer's instructions.

### Transmission Electron Microscopy

Kidney cortex and HKC‐8 cells were fixed in 1.25% glutaraldehyde (0.1 mol/L). Ultrathin sections (60 nm) were prepared 54 and were examined under an electron microscope (JEOL JEM-1010, Tokyo, Japan).

### ATP, Complex I Activity, and JC-1 Analysis

ATP production was tested using enhanced ATP assay kit (Beyotime Biotechnology**,** S0027). Complex I activity was determined using a commercial kit (Abcam, ab109721). The mitochondrial membrane potential was determined by JC-1 staining accordingly 27.

### RNA-seq analysis

RNA-seq was performed to characterize the transcriptome of groups of mice treated with UIRI or UIRI/PA-S14. Total RNA was isolated using Trizol reagent (Invitrogen, Carlsbad, CA). cDNA libraries were constructed and assessed on the Agilent Bioanalyzer 2100 system, and sequenced at the Novogene Bioinformatics Institute (Beijing, China) on an Illumina Novaseq platform. Index of the reference genome was built using Hisat2 v2.0.5 and paired-end clean reads were aligned to the reference genome using Hisat2 v2.0.5. Genes with an adjusted *P*-value <0.05 were assigned as differentially expressed.

### Surface Plasmon Resonance Imaging (SPRi) Assay

The SPR analyses were performed on a PlexArray HT A100 system (Plexera LLC, Bothell, WA, USA). Recombinant Human LKB1 (CSB-EP624036HU; Cusibio technology, WuHan, China) was immobilized on a bare gold SPRi chip (3D Dextran chip). The SPRi chip was blocked using 1 M ethanolamine aqueous solution (pH=8.5). Samples of PA-S14 (purity >95%) were diluted to indicated concentrations. Data were fitted to a 1:1 binding model to obtain KD by a PlexArray HT A100 (plexera) system. Data were analyzed using BIAevaluation Software.

### Statistical analyses

All data examined were expressed as mean ± SEM. Statistical analysis of the data was carried out using SPSS 20.0 (SPSS Inc.). Comparison between groups was made using one-way ANOVA followed by Student-Newman-Kuels test or Dunnett's T3 procedure. *P* < 0.05 was considered to represent a significant difference.

## Translational statement

LKB1 is the key regulator in energy metabolism. Loss of function of LKB1 is involved in the pathogenesis of renal fibrosis. However, LKB1 activators are not available in commercial. This study provides a new natural product compound, PA-S14, which serves as LKB1 activator. PA-S14 strongly induces LKB1/AMPK signaling, triggers autophagy and mitochondrial biogenesis. Importantly, PA-S14 effectively protects against tubular cell senescence and renal fibrosis. The finding provides a new therapeutic potential to renal fibrosis and the dysfunction of energy metabolism.

## Supplementary Material

Supplementary materials and methods, figures.
