# Supplementary material for: A new LKB1 activator, piericidin analogue S14, retards renal fibrosis through promoting autophagy and mitochondrial homeostasis in renal tubular epithelial cells
Source: Theranostics. 2022 Oct 9;12(16):7158–79. doi: 10.7150/thno.78376 (PMC9576617; doi:10.7150/thno.78376)
Supplement: Supplementary file 1 — Supplementary materials and methods, figures. [file thnov12p7158s1.pdf]

Supplementary Material

A new LKB1 activator, piericidin analogue S14, retards renal fibrosis through promoting autophagy and mitochondrial homeostasis in renal tubular epithelial cells

Canzhen Liu [1#], Xiaoxu Wang [1#], Xiaonan Wang [1#], Yunfang Zhang [2#], Wenjian Min [3], Ping Yu [4], Jinhua Miao [1], Weiwei Shen [1], Shuangqin Chen [1], Shan Zhou [1], Xiaolong Li [1], Ping Meng [2], Qinyu Wu [1], Fan Fan Hou [1], Youhua Liu [1], Peng Yang [3], Cheng Wang [5*], Xu Lin [6*], Lan Tang [4*], Xuefeng Zhou [7*], Lili Zhou [1*]

[1] Division of Nephrology, Nanfang Hospital, Southern Medical University; National Clinical Research Center for Kidney Disease; State Key Laboratory of Organ Failure Research; Guangdong Provincial Institute of Nephrology; Guangdong Provincial Key Laboratory of Renal Failure Research, Guangzhou, China.; [2] Department of Nephrology, Huadu District People's Hospital, Southern Medical University, Guangzhou, China; [3] State Key Laboratory of Natural Medicines and Jiang Su Key Laboratory of Drug Design and Optimization, China Pharmaceutical University, Nanjing, China; [4] Guangdong Provincial Key Laboratory of New Drug Screening, School of Pharmaceutical Sciences, Southern Medical University, Guangzhou, China; [5] Division of nephrology, Department of medicine, the Fifth affiliated hospital Sun Yat-Sen University, Zhuhai, Guangdong, China; [6] Department of Nephrology, The Affiliated Hospital of Youjiang Medical University for Nationalities, Baise, Guangxi, China; [7] CAS Key Laboratory of Tropical Marine Bio-resources and Ecology, Guangdong Key Laboratory of Marine Materia Medica, South China Sea Institute of Oceanology, Chinese Academy of Sciences,

Guangzhou, China.

# These authors contributed equally to this work.

Short Title: Piericidin analogue S14 inhibits renal fibrosis through LKB1 activation

*Corresponding author:

Dr. Lili Zhou, Division of Nephrology, Nanfang Hospital, 1838 North Guangzhou Ave, Guangzhou 510515, China, E-mail: jinli730@smu.edu.cn; or Dr. Xuefeng Zhou, Guangdong Key Laboratory of Marine Materia Medica, South China Sea Institute of Oceanology, Chinese Academy of Sciences, Guangzhou, China, E-mail: xfzhou@scsio.ac.cn; or Dr. Lang Tang, School of Pharmaceutical Sciences, Southern Medical University, Guangzhou, 510515, China, E-mail: tl405@smu.edu.cn; or Dr. Xu Lin, The Affiliated Hospital of Youjiang Medical University for Nationalities, Baise, Guangxi, China, E-mail: linyyfyy@163.com; or Dr. Cheng Wang, the Fifth affiliated hospital Sun Yat-Sen University, Zhuhai, Guangdong, China, E-mail: wangch2@mail.sysu.edu.cn.

Supplementary Detailed Methods

PA and PA-S14

The strain *Streptomyces psammoticus* SCSIO NS126, isolated from a mangrove sediment sample collected from the Pearl River estuary to South China Sea, was fermented for 100 L, according to a previously described method [1]. The culture broth was extracted with ethyl acetate three times, and then concentrated under vacuum. The extract (75.0 g) was chromatographed on silica gel to give eight fractions (Frs.1~8). Frs.2 was purified by silica gel to obtain a pure compound (355 mg), which was identified as piericidin A (PA) as reported in our previous study [1]. With the guide of HPLC analysis, Frs.7 was purified by silica gel again to give six sub-fractions (Frs.7-1~7-6). Frs.7-4 was purified by semipreparative HPLC to obtain a piericidin analogue compound labeled as S14 (PA-S14, 52 mg). PA-S14 was suggested to be a hydroxylative product of glucopiericidin A because of its molecular weight 593 given by MS analysis (Figure S2). It was determined to be 13-hydroxyglucopiericidin A by comparison of its ^{1}H and ^{13}C NMR date (Figure S2) with literature data [2]. The obtained compounds, determined to have ≥95% purity by analytical HPLC, were stored at -20 ℃ until use and dissolved in DMSO to a stock concentration of 10 mM.

Animal models

Mouse models of renal fibrosis were constructed, including ADR, UIRI, UUO and 5/6NX, as described previously [3, 4]. Male BALB/c or CD-1 mice (weighing 20-25g) were obtained from Southern Medical University Animal Center, Guangzhou, China, and housed in the standard environment with regular light/dark cycles and free access to water and chow diet.

For ADR model, BALB/c mice were administered a single intravenous injection of ADR (doxorubicin hydrochloride; Sigma, St. Louis, MO) at 11 mg/kg body weight. Mice were randomly assigned to one of four groups (n = 5): 1) control, 2) ADR + vehicle control, 3) ADR + PA-S14 (0.5 mg/kg body weight), and 4) ADR + PA-S14 (1.0 mg/kg body weight). At 2 weeks after ADR injection, mice were intraperitoneally

injected with PA-S14 at 0.5 or 1.0 mg/kg/d for 7 days. Mice were euthanized at 3 weeks after ADR injection.

For UIRI model, BALB/c mice were subjected to unilateral ischemia-reperfusion (IRI) surgery by an established protocol as described previously [3]. Briefly, left renal pedicles were clipped for 35 minutes using microaneurysm clamps (item no.18051–35; Fine Science Tools, Cambridge, UK) for IRI injury. After removing clamps, reperfusion was visually confirmed. During the ischemic period, body temperature was maintained at approximately 37 ℃~38 ℃ using a temperature-controlled heating system. Mice were randomly assigned to one of three groups (n = 5): 1) sham control, 2) UIRI + vehicle control, and 3) UIRI + PA-S14 (1.0 mg/kg/d). At 4 days after IRI, mice were subjected to daily intraperitoneal injections of PA-S14 for 7 days. After 10 days post-IRI, the intact right kidney was removed via a right flank incision. Mice were sacrificed at 11 days post-IRI, respectively.

For UUO model, BALB/c mice were carried out by double-ligating the left ureter following a midline abdominal incision. Mice were randomly assigned to one of three groups (n = 5): 1) sham control, 2) UUO + vehicle control, and 3) UUO + PA-S14 (1.0 mg/kg body weight). Another set of experiments was performed, in which PA-S14 was given for 3 times (2, 4, 6 days after UUO) via intraperitoneal injections at 1.0 mg/kg body weight. Mice were euthanized at 7 days after UUO.

For the 5/6NX model, male CD-1 mice were randomly divided into three group(n = 5)： 1) sham control, 2) 5/6NX + vehicle control, and 3) 5/6NX + PA-S14 (1.0 mg/kg body weight). Briefly, the general operation is as follows. The upper and lower poles of the left kidney, two thirds of the left kidney, were removed. The remnant left kidney was immediately compressed using gelatin sponge to stop bleeding (about 30s). One week later, the right renal vessels were ligated and the right kidney was removed (week 0). At 4 weeks after operation, mice were subjected to daily intraperitoneal injections of PA-S14 for 2 weeks. All mice were euthanized and sacrificed at week 6.

In some experiments, the healthy BALB/c mice were also treated with PA-S14 at

the dosage of 1.0 mg/kg via intraperitoneal injection. Mice were randomly assigned to one of two groups (n = 5): 1) vehicle control mice, 2) PA-S14 mice (1mg/kg body weight). Mice were euthanized at 7 days after PA-S14 injection.

All animal studies were performed adhering to the Health Guide for the Care and Use of Laboratory Animals and approved by the Experimental Animal Committee at the Nanfang Hospital, Southern Medical University (NFYY-2017-0425).

Urinary albumin, serum creatinine and BUN assay

Urinary albumin was measured using a mouse albumin enzyme-linked immunosorbent assay quantitation kit according to the manufacturer's protocol (Bethyl Laboratories, Montgomery, TX). Serum creatinine and BUN levels were determined by an automatic chemistry analyzer (AU480 Chemistry Analyzer, Beckman Coulter, Atlanta, Georgia). Urinary albumin was standardized to urine creatinine and expressed as mg/mg urine creatinine. The levels of serum creatinine and BUN were expressed as mg/dl.

Cell culture and treatment

Human proximal tubular epithelial cells (HKC-8) were provided by Dr. Lorraine C. Racusen (Johns Hopkins University, Baltimore, MD), and cultured as described previously [6]. Cells were pretreated with 0.5 μM of PA-S14 for 1 h, and treated with human recombinant TGF-β1 (5 ng/ml; R&D Systems, 240-B-010), PP2Cα (2.2 μg/ml; R&D Systems, 4150-PP) or autophagy inhibitor Bafilomycin A1 (100 nM; Abcam, ab120497). HKC-8 cells were also transfected with empty vector (pcDNA3), LKB1 wild type and mutated plasmids (gene create, Wuhan, China), or human LKB1 siRNA using Lipofectamine 2000 transfection reagent (Thermo, 11668019). LKB1 siRNA (si-LKB1) sequence and the corresponding negative control (si-NC) were described as previously reported [7].

MTT assay

Cell viability was assessed by the MTT

(3-(4,5-dimethylthiazol-2-yl)-2,5-diphenyl-tetrazolium bromide) assay, as described previously [8].

Detection of Autophagic Flux

LC3 Puncta accumulation

HKC-8 cells were transfected with pCMV-GFP-LC3B plasmid (Beyotime Biotechnology, D2815, Beijing, China). GFP-LC3B exists in the cytoplasm in the form of dispersion without autophagy; In the case of autophagy, GFP-LC3B aggregates on the autophagosome membrane in the form of spots (LC3B dots or punctae).

mRFP-GFP-LC3B lentivirus infection

Autophagic flux was monitored using the mRFP-GFP-LC3B lentivirus (Hanbio Biotechnology, HB-LP210) based on different pH stabilities of RFP and GFP. HKC-8 cells were infected with lentiviruses expressing a tandem RFP-GFP-LC3B fusion protein and were then treated with TGF-β1 or were cotreated with PA-S14. After different treatments, the cells were observed under the confocal microscope. Autophagosomes exhibit yellow dots (RFP and GFP signal), whereas autophagolysosomes exhibit red dots (RFP signal).

Western blot analysis

Protein samples were prepared and loaded onto SDS-PAGE gel to running. When the running finished, protein samples were electrotransfered onto PVDF membrane. PVDF membrane was then incubated in 5% of milk for one hour at room temperature, and incubated with different primary antibodies overnight at 4°C. The next day, PVDF membrane was incubated with a responding secondary antibody for one hour at room temperature. Chemiluminescent detection was performed. The whole process was performed as described previously [6]. The primary antibodies used were as follows: Fibronectin (Sigma-Aldrich, F3648), α-SMA (Abcam, ab5694), Collagen I (Boster Biological Technology, BA0325), KIM-1 (Boster Biological Technology, BA3537),

Klotho (R&D Systems, AF1819), PGC-1α (Abcam, ab54481), TFAM (Boster Biological Technology, PB0413), TOMM20 (Abcam, ab186735), LC3B (Abcam, ab48394), p62/SQSTM1 (Abcam, ab109012), ATG5 (Boster Biological Technology, BA3525-2), mTOR (Abcam , ab2732), p-mTOR (Abcam, ab109268), STRAD (Abcam, ab192879), CAB39/M025(Abcam, ab51132), p-AMPKα (Cell Signaling Technology, 2535S), AMPKα (Cell Signaling Technology, 2532S), p-LKB1 (Cell Signaling Technology, 3482S), LKB1 (Santa Cruz Biotechnology, sc-32245), active-β-catenin (Cell Signaling Technology, 19807S), $p16^{INK4A}$(Abcam, ab189034), $p14^{ARF}$ (Abcam, ab185620), PAI-1 (R&D Systems, AF1819), p-MARK(Cell Signaling Technology, 2532S), MARK2(Cell Signaling Technology, 9118S), p-SIK(Abmart, TA8189S), SIK(Proteintech，51045-1-AP), PP2C-α (Cell Signaling Technology, 3549S), GAPDH (Ray Antibody Biotech, RM2002), α-tubulin (Ray Antibody Biotech, RM2007), and β-actin (Ray Antibody Biotech, RM2001).

qRT-PCR

Total RNA was obtained using a TRIzol RNA isolation system (Life Technologies, Grand Island, NY). Real-time PCR was performed on an ABI PRISM 7000 Sequence Detection System (Applied Biosystems, Foster City, CA). The sequences of the primer pairs used in qRT-PCR were as follows: mouse STK11, 5'-TTGGGCCTTTTCTCCGAGG-3' and 5'-CAGGTCCCCCATCAGGTACT-3'; mouse Prkaa1 (AMPKα1), 5'-GTCAAAGCCGACCCAATGATA-3' and 5'-CGTACACGCAAATAATAGGGGTT-3'; mouse NGAL, 5'-CTTGATCCCTGCCCCATCTC-3' and 5'-ACATCGTAAAGCTGCCTTCTG-3'; mouse Fibronectin , 5’-GATGAGCTTCCCCAACTGGT-3' and 5'-CTGGGTTGTTGGTGGGATGT-3'; mouse β-actin, 5’-AGGCATCCTCACCCTGAAGTA-3’ and 5’-CACACGCAGCTCATTGTAGA-3’; Human STK11, 5’-TGTCGGTGGGTATGGACAC-3’ and 5’-CCTTGCCGTAAGAGCCTTCC-3’; Human β-actin, 5’-CTCACCATGGATGATGATATCGC-3’ and 5’-AGGAATCCTTCTGACCCATGC-3’.

Histology and immunohistochemical staining

Paraffin-embedded kidney sections were performed with periodic acid-Schiff (PAS) and Sirius red staining to identify injured tubules and collagen deposition. The lesions were quantified by a computer-aided technique [9]. Immunohistochemical staining was performed as described previously [6]. Antibodies used were as follows: p-LKB1 (Cell Signaling Technology, 3482S), p-AMPKα (Cell Signaling Technology, 2535S), TOMM20 (Abcam, ab186735), KIM-1 (R&D Systems, AF1817), Fibronectin (Sigma-Aldrich, F3648), α-SMA (Abcam, ab5694;), Klotho (R&D Systems, AF1819), PGC-1α (Abcam, ab54481), p62/SQSTM1 (Abcam, ab109012), p-mTOR (Abcam, ab109268).

Immunofluorescence staining

Kidney cryosections (3 μm) were fixed with 4% paraformaldehyde for 15 min at room temperature. HKC-8 cells cultured on coverslips were fixed with cold methanol:acetone (1:1) for 15 min at room temperature. After blocking with 10% donkey serum for 60 min, the slides were immunostained with primary antibodies against Lotus Tetragonolobus Lectin (LTL) (VECTOR Laboratories, FL-1321), Peanut Agglutinin (PNA) (VECTOR Laboratories, FL-1071), and Dolichos Biflorus Agglutinin (DBA) (VECTOR Laboratories, FL1031), Fibronectin (Sigma Aldrich, F3648), TOMM20 (Abcam, ab186735), LC3B (Abcam, ab128025), p-AMPKα (Cell Signaling Technology, 2535S), SQSTM1(Abcam, ab207305), PGC-1α (Abcam, ab54481). The slides were then stained with Cy3- or Cy2-conjugated secondary antibody (Jackson ImmunoResearch Laboratories), and mounted with Vectashield anti-fade mounting media by using DAPI to visualize the nuclei (Vector Laboratories, Burlingame, CA). Images were taken with fluorescence microscopy (Leica DMi8; Leica Microsystems) or a Leica TCS-SP8 confocal microscope.

SA-β-gal, mitoSOX, and MitoTracker staining

Frozen sections (3 μm) were performed β-galactosidase activity staining (Cell

Signaling Technology, 9860S), MitoTracker deep red (Thermo Fisher, M22426), and mitoSOX (Thermo Fisher, M36008) staining to detect senescence, mitochondria mass and mitochondrial ROS production, respectively. Cultured cells were stained for β-galactosidase activity, MitoTracker deep red, and mitoSOX detection according to the manufacturer's instructions.

Adenoviral transfection

HKC-8 cells were plated in 6-well plates and reached 50% confluence at the time of transfection. Then, cells were transfected with adenoviral vectors expressing LKB1 siRNA (si-LKB1) at MOI of 40 which were purchased from HanBio Technology (Shanghai, China) and performed according to the manufacturer's instructions. After 24h transfection, the medium containing the adenovirus was removed and replaced with fresh medium. Meanwhile, treated with TGF-β1 or cotreated with PA-S14 for 24 h. After different treatments, cells were then collected and subjected to analyses.

Transmission Electron Microscopy

To assess mitochondrial morphology and autophagic vacuoles, kidney cortex and HKC-8 cells were collected and fixed in 1.25% glutaraldehyde (0.1 mol/L) in phosphate buffer. Ultrathin sections (60 nm) were prepared by a routine procedure [10] and were examined under an electron microscope (JEOL JEM-1010, Tokyo, Japan).

ATP assay

ATP concentrations were tested using enhanced ATP assay kit (Beyotime Biotechnology, S0027) according to the manufacturer's protocol.

Complex I Activity Assay

Complex I activity was determined using a commercial kit (Abcam, ab109721) according to the procedures specified by the manufacturer.

JC-1 Analysis

The mitochondrial membrane potential was determined by JC-1 staining and operated according to the previous literature [11].

RNA-seq analysis

RNA-seq was performed to characterize the transcriptome of groups of mice treated with UIRI or UIRI/PA-S14. Trizol reagent (Invitrogen, Carlsbad, CA) was used to isolate the total RNA of each sample. RNA integrity was assessed using the RNA Nano 6000 Assay Kit of the Bioanalyzer 2100 system (Agilent Technologies, CA, USA). Then, a total of six cDNA libraries were constructed. PCR products were purified (AMPure XP system) and library quality was assessed on the Agilent Bioanalyzer 2100 system. The clustering of the index-coded samples was performed on a cBot Cluster Generation System using TruSeq PE Cluster Kit v3-cBot-HS (Illumia). After cluster generation, the libraries were sequenced at the Novogene Bioinformatics Institute (Beijing, China) on an Illumina Novaseq platform and 150 bp paired-end reads were generated. Index of the reference genome was built using Hisat2 v2.0.5 and paired-end clean reads were aligned to the reference genome using Hisat2 v2.0.5. FPKM, expected number of Fragments Per Kilobase of transcript sequence per Millions base pairs sequenced, is currently the most commonly used method for estimating gene expression levels. Gene Set Enrichment Analysis (GSEA) is a computational approach to determine if a predefined Gene Set can show a significant consistent difference between two biological states. Genes with an adjusted P-value <0.05 were assigned as differentially expressed. The transcriptomics analysis of kidneys from control and PA-S14-treated mice was completed by Shanghai applied protein technology, and the detailed operation steps are similar to the above.

Surface Plasmon Resonance Imaging (SPRi) Assay.

The SPR analyses were performed on a PlexArray HT A100 system (Plexera LLC, Bothell, WA, USA). Recombinant Human Serine/threonine-protein kinase STK11 (LKB1) (64.3 kDa, >90% as determined by SDS-PAGE) (CSB-EP624036HU;

Cusibio technology, WuHan, China) diluted at 1mg/ml in PBS (PH=7.4), was immobilized on a bare gold SPRi chip (3D Dextran chip). Then, the chip was incubated in 4 ℃ overnight with more than 60% humidity. The SPRi chip was washed three times with PBS and blocked by using 1 M ethanolamine aqueous solution (pH=8.5). Samples of PA-S14 (purity >95%) as the analyte were diluted with PBS to concentrations of 0.5, 5, 25, and 50μM. Data were fitted to a 1:1 binding model to obtain KD. All the samples were tested by a PlexArray HT A100 (plexera) system, and data were analyzed using BIAevaluation Software.

Statistical analyses

All data examined were expressed as mean ± SEM. Statistical analysis of the data was carried out using SPSS 20.0 (SPSS Inc.). Comparison between groups was made using one-way ANOVA followed by Student-Newman-Kuels test or Dunnett's T3 procedure. $P < 0.05$ was considered to represent a significant difference.

References

1. Zhou XF, Liang Z, Li KL, Fang W, Tian YX, Luo XW, et al. Exploring the natural piericidins as anti-renal cell carcinoma agents targeting peroxiredoxin 1. J Med Chem. 2019; 62: 7058-69.
2. Mori H, Funayama S, Sudo Y, Komiyama K, Omura S. A new antibiotic, 13-hydroxyglucopiericidin A. Isolation, structure elucidation and biological characteristics. J Antibiot (Tokyo). 1990; 43: 1329-31.
3. Xiao LX, Zhou D, Tan RJ, Fu HY, Zhou LL, Hou FF, et al. Sustained activation of Wnt/beta-Catenin signaling drives AKI to CKD progression. J Am Soc Nephrol. 2016; 27: 1727-40.
4. Zhou LL, Li YJ, Hao S, Zhou D, Tan RJ, Nie J, et al. Multiple genes of the renin-angiotensin system are novel targets of Wnt/beta-catenin signaling. J Am Soc Nephrol. 2015; 26: 107-20.
5. Zhao X, Ho D, Gao S, Hong C, Vatner DE, Vatner SF. Arterial pressure monitoring in mice. Current protocols in mouse biology. 2011; 1: 105-22.
6. Zhou D, Tian Y, Sun L, Zhou LL, Xiao LX, Tan RJ, et al. Matrix metalloproteinase-7 Is a urinary biomarker and pathogenic mediator of kidney fibrosis. J Am Soc Nephrol. 2017; 28: 598-611.
7. Zeng J, Liu W, Fan YZ, He DL, Li L. PrLZ increases prostate cancer docetaxel resistance by inhibiting LKB1/AMPK-mediated autophagy. Theranostics. 2018; 8: 109-23.
8. Zhou D, Li YJ, Zhou LL, Tan RJ, Xiao LX, Liang M, et al. Sonic hedgehog is a novel tubule-derived growth factor for interstitial fibroblasts after kidney injury. J Am Soc Nephrol. 2014; 25: 2187-200.

9. Rosano L, Cianfrocca R, Tocci P, Spinella F, Di Castro V, Spadaro F, et al. beta-arrestin-1 is a nuclear transcriptional regulator of endothelin-1-induced beta-catenin signaling. Oncogene. 2013; 32: 5066-77.

10. Xiao L, Xu XX, Zhang F, Wang M, Xu Y, Tang D, et al. The mitochondria-targeted antioxidant MitoQ ameliorated tubular injury mediated by mitophagy in diabetic kidney disease via Nrf2/PINK1. Redox Biol. 2017; 11: 297-311.

11. Miao JH, Liu JF, Niu J, Zhang YF, Shen WW, Luo CW, et al. Wnt/beta-catenin/RAS signaling mediates age-related renal fibrosis and is associated with mitochondrial dysfunction. Aging Cell. 2019; 18: e13004.

Supplementary Figures

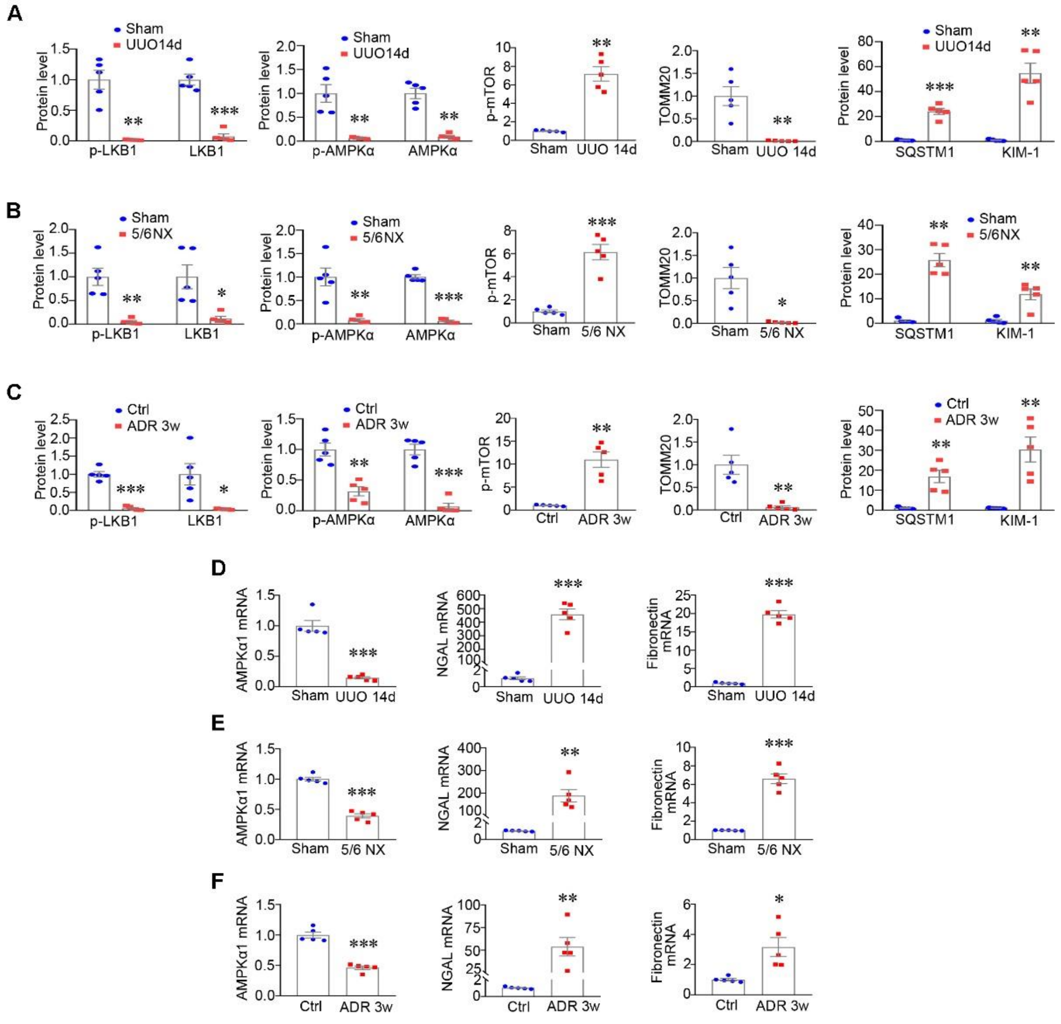

Supplementary Figure S1

Supplementary Figure S1. Quantitative data for LKB1/AMPK signaling proteins or genes expression in different CKD models are shown. (**A-C**) Quantitative data for Figure 1A, C, E. **P* < 0.05, ***P* < 0.01, ****P* < 0.001 versus sham controls (n = 5). (**D-F**) Quantitative PCR results showing renal expression of AMPKα1, NGAL and Fibronectin in different models of CKD as indicated. **P* < 0.05, ***P* < 0.01, ****P* < 0.001 versus control mice (n = 5).

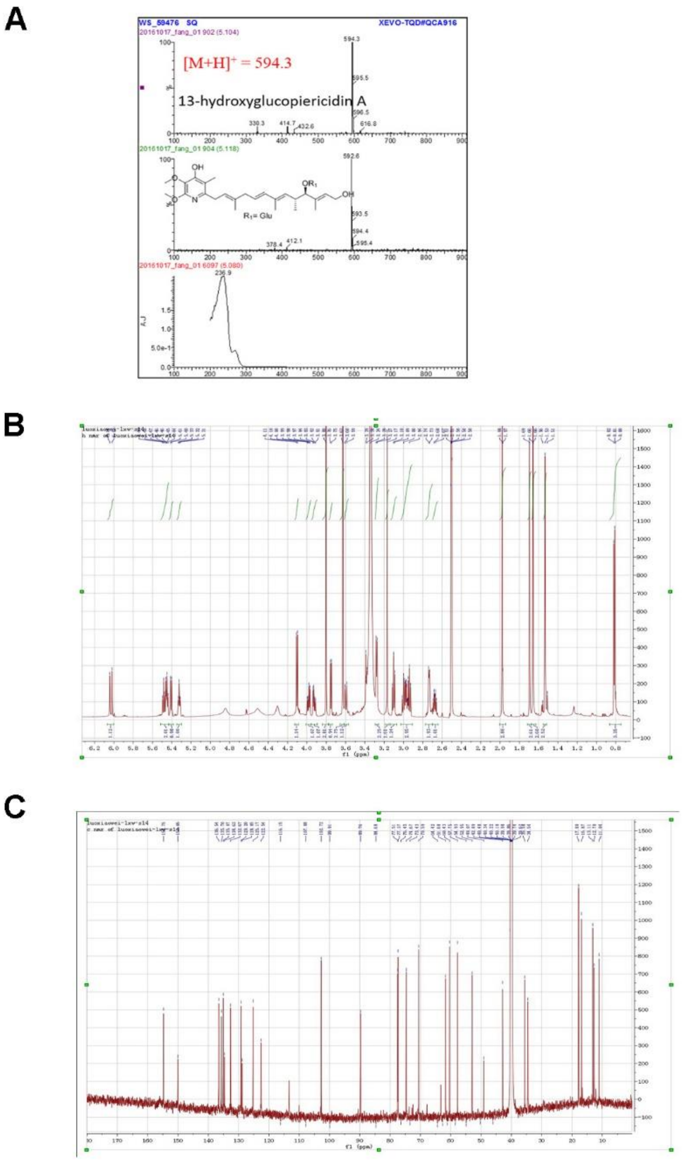

Supplementary Figure S2

Supplementary Figure S2. MS, UV, ^{1}H NMR, and ^{13}C NMR Spectrum of PA-S14.

(**A**) MS and UV spectrum of PA-S14. (**B**) ^{1}H NMR spectrum of **PA-S14** (in DMSO-d_6, 700 MHz). (**C**) ^{13}C NMR spectrum of PA-S14 (in DMSO-d_6, 175 MHz).

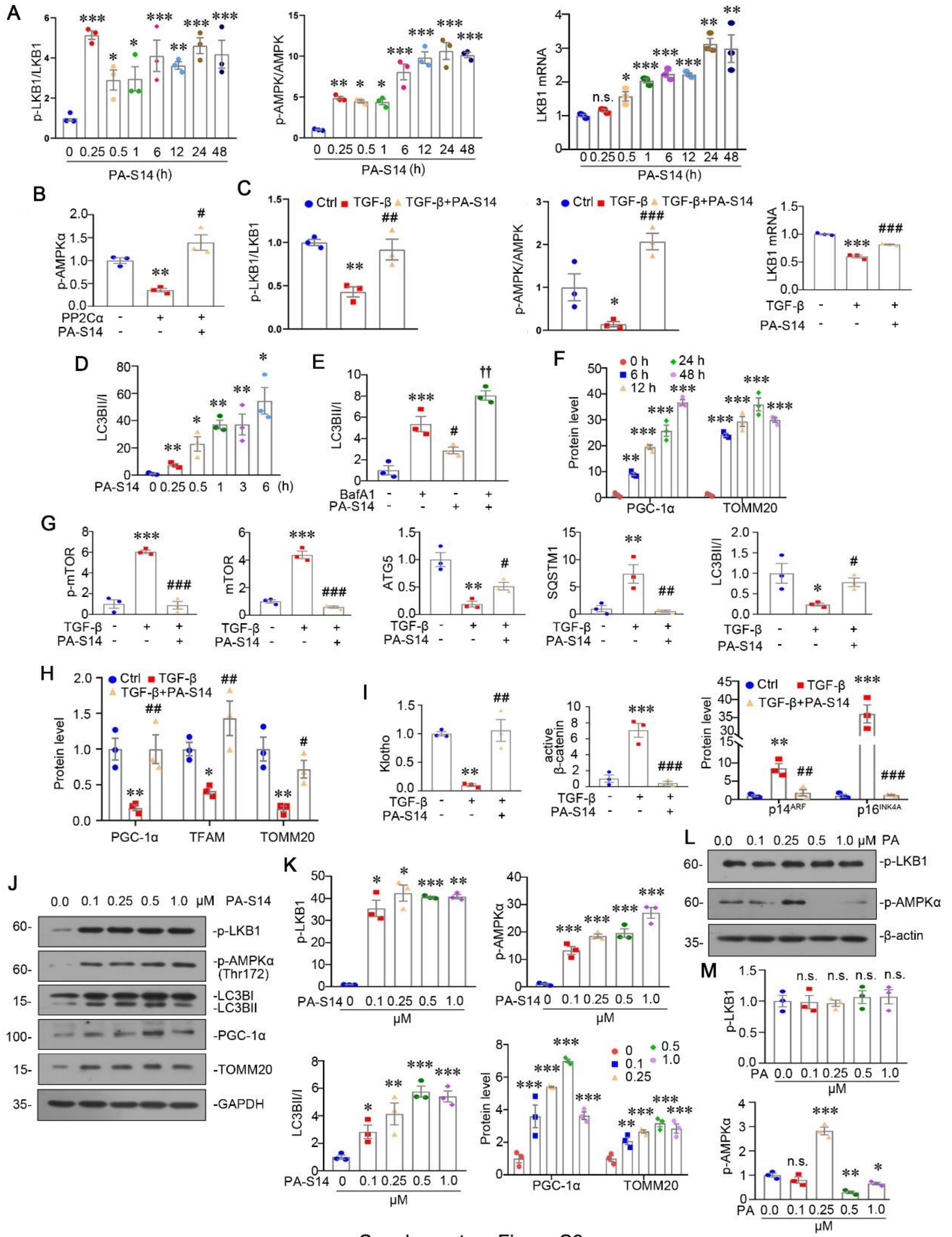

Supplementary Figure S3

Supplementary Figure S3. PA-S14 induces LKB1/AMPK signaling in vitro. (**A**) Quantitative data of the ratio of p-LKB1/total LKB1 and p-AMPK/total AMPK for Figure 2M. **P* < 0.05, ***P* < 0.01, ****P* < 0.001 versus control group (n = 3). Quantitative PCR result showing PA-S14 time-dependently induced the upregulation

of LKB1 mRNA. *$P < 0.05$, **$P < 0.01$, ***$P < 0.001$ versus control group (n = 3). (**B**) Quantitative data for Figure 2N. **$P < 0.01$ versus control group; #$P < 0.05$ versus PP2Cα group (n = 3). (**C**) Quantitative data of the ratio of p-LKB1/total LKB1 and p-AMPK/total AMPK for Figure 2O. *$P < 0.05$, **$P < 0.01$ versus control group; ##$P < 0.01$, ###$P < 0.001$ versus TGF-β1 group (n = 3). Quantitative PCR result shows the relative abundance of LKB1 mRNA in different groups. ***$P < 0.001$ versus control group; ###$P < 0.001$ versus TGF-β1 group (n = 3). (**D**) Quantitative data for Figure 3A. Graphic presentations of LC3BII/I ratio in different groups as indicated. *$P < 0.05$, **$P < 0.01$versus control group (n = 3). (**E**) Quantitative data for Figure 3B. ***$P < 0.001$ versus control group; #$P < 0.05$ versus control group; ††$P < 0.01$versus the BafA1 group alone (n = 3). (**F**) Quantitative data for Figure 3D. **$P < 0.01$, ***$P < 0.001$ versus control group (n = 3). (**G**) Quantitative data for Figure 3F. *$P < 0.05$, **$P < 0.01$, ***$P < 0.001$ versus control group; #$P < 0.05$, ##$P < 0.01$, ###$P < 0.001$ versus TGF-β1 group (n = 3). (**H**) Quantitative data for Figure 3J. *$P < 0.05$, **$P < 0.01$ versus control group; #$P < 0.05$, ##$P < 0.01$ versus TGF-β1 group (n = 3). (**I**) Quantitative data for Figure 3M and N. **$P < 0.01$, ***$P < 0.001$ versus control group; ##$P < 0.01$, ###$P < 0.001$ versus TGF-β1 group (n = 3). (**J-K**) The expression levels of p-LKB1, p-AMPKα, LC3BII/LC3BI ratio, PGC-1α and TOMM20 were detected by western blotting in HKC-8 cells following treatment with different doses of PA-S14 (0, 0.1, 0.25, 0.5, 1.0 μM) for 24 h. *$P < 0.05$, **$P < 0.01$, ***$P < 0.001$ versus control group (n = 3). (**L-M**) The expression levels of p-LKB1 and p-AMPKα were assayed by western blotting in HKC-8 cells following treatment

with different doses of PA (0, 0.1, 0.25, 0.5, 1.0 μM) for 24 h. Graphic presentations of p-LKB1 and p-AMPKα proteins in different groups as indicated. *P < 0.05, **P < 0.01, ***P < 0.001 versus control group (n = 3).

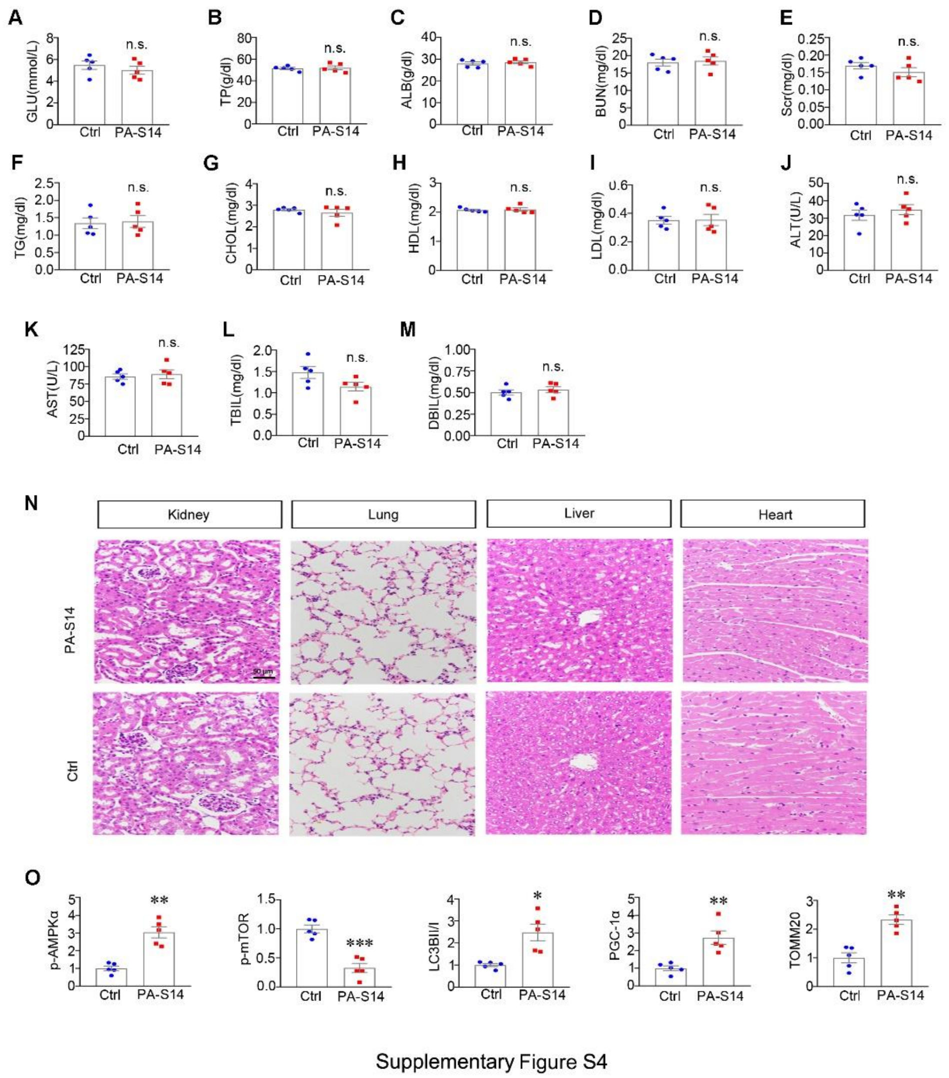

Supplementary Figure S4. **PA-S14 exhibits no adverse side-effect on normal mice.** (**A-M**) Quantitative data for blood biochemical indexes in control group and PA-S14 group. (**N**) Representative staining micrographs of HE staining in multiple organs in two groups. Paraffin sections were performed with HE staining. Scale bar, 50 μm. (**O**) Quantitative data for Figure 4J. *P < 0.05, **P < 0.01, ***P < 0.001 versus control

mice (n = 5).

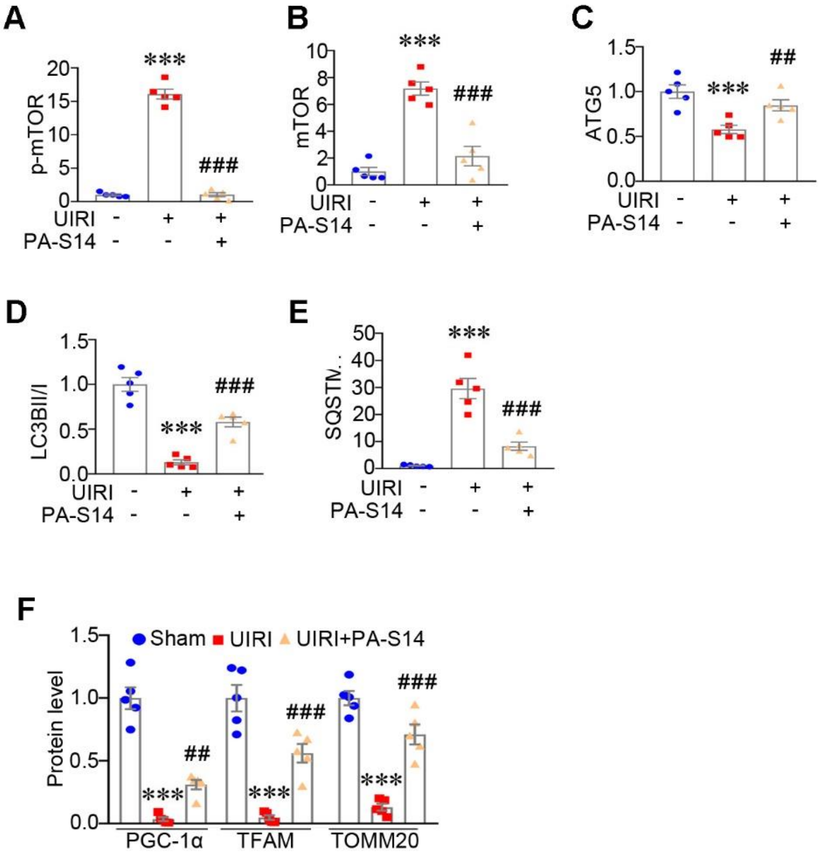

Supplementary Figure S5

Supplementary Figure S5. PA-S14 triggers autophagy and preserves mitochondrial biogenesis in UIRI mice. (**A-F**) Quantitative data for Figure5H and J. ****P* < 0.001 versus sham controls; ##*P* < 0.01, ###*P* < 0.001 versus UIRI (n = 5).

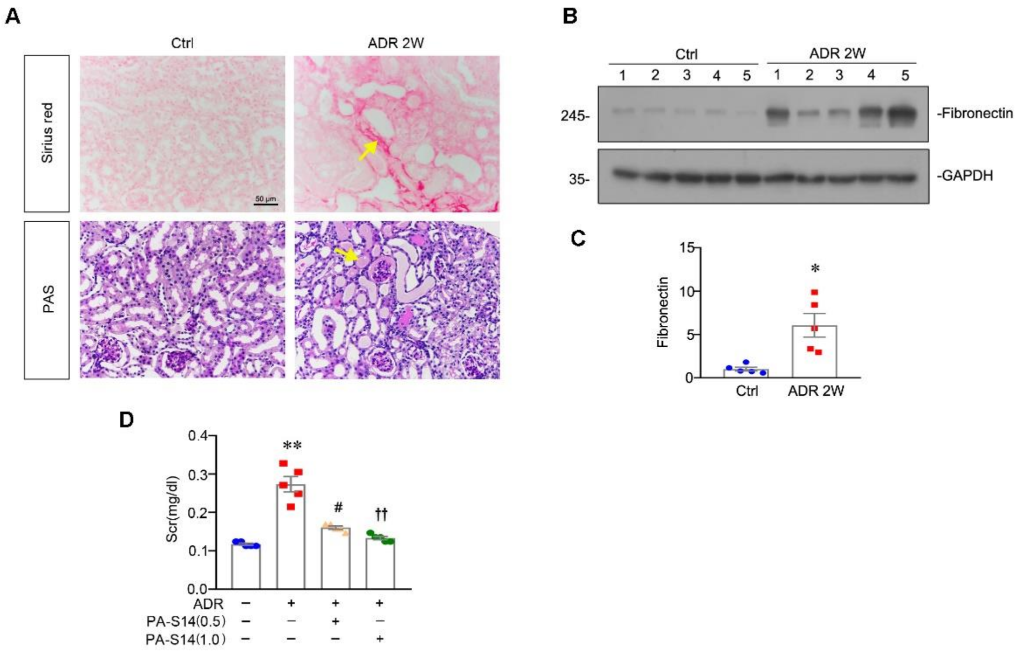

Supplementary Figure S6

Supplementary Figure S6. Renal fibrosis is established in mice injected with adriamycin for 2 weeks. (**A**) Paraffin kidney sections from the mice injected with adriamycin (ADR) for 2 weeks were subjected to Sirius red staining. Arrow indicates the deposition of collagen. Scale bar, 50 µm. Paraffin kidney sections from the mice injected with ADR for 2 weeks were subjected to Periodic acid–Schiff (PAS) staining. Arrow indicates the damaged tubules. Scale bar, 50 µm. (**B-C**) Representative western blot and quantitative data show renal expression of Fibronectin in mice treated with ADR for 2 weeks. Numbers (1-5) indicate each individual animal in given group. **P* < 0.05 versus control mice. (**D**) Serum creatinine levels in different groups as indicated. Serum creatinine was expressed as milligrams per deciliter. ***P* < 0.01 versus control mice; #*P* < 0.05 versus ADR mice; ††*P* < 0.01 versus ADR mice (n = 5).

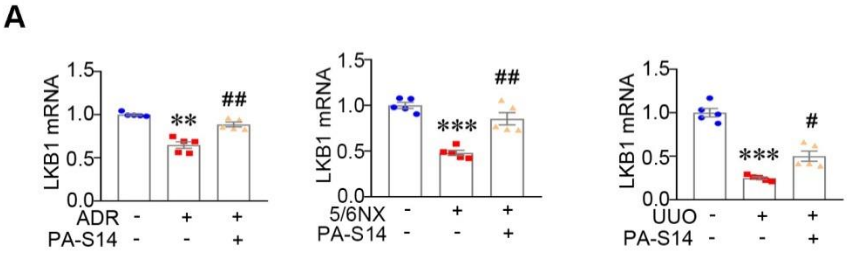

Supplementary Figure S7

Supplementary Figure S7. PA-S14 upregulated the mRNA levels of LKB1 in different mouse models. (**A**) Quantitative PCR results showing renal expression of LKB1. ***P* < 0.01, ****P* < 0.001 versus control group; #*P* < 0.05, ##*P* < 0.01versus model group (n = 5).

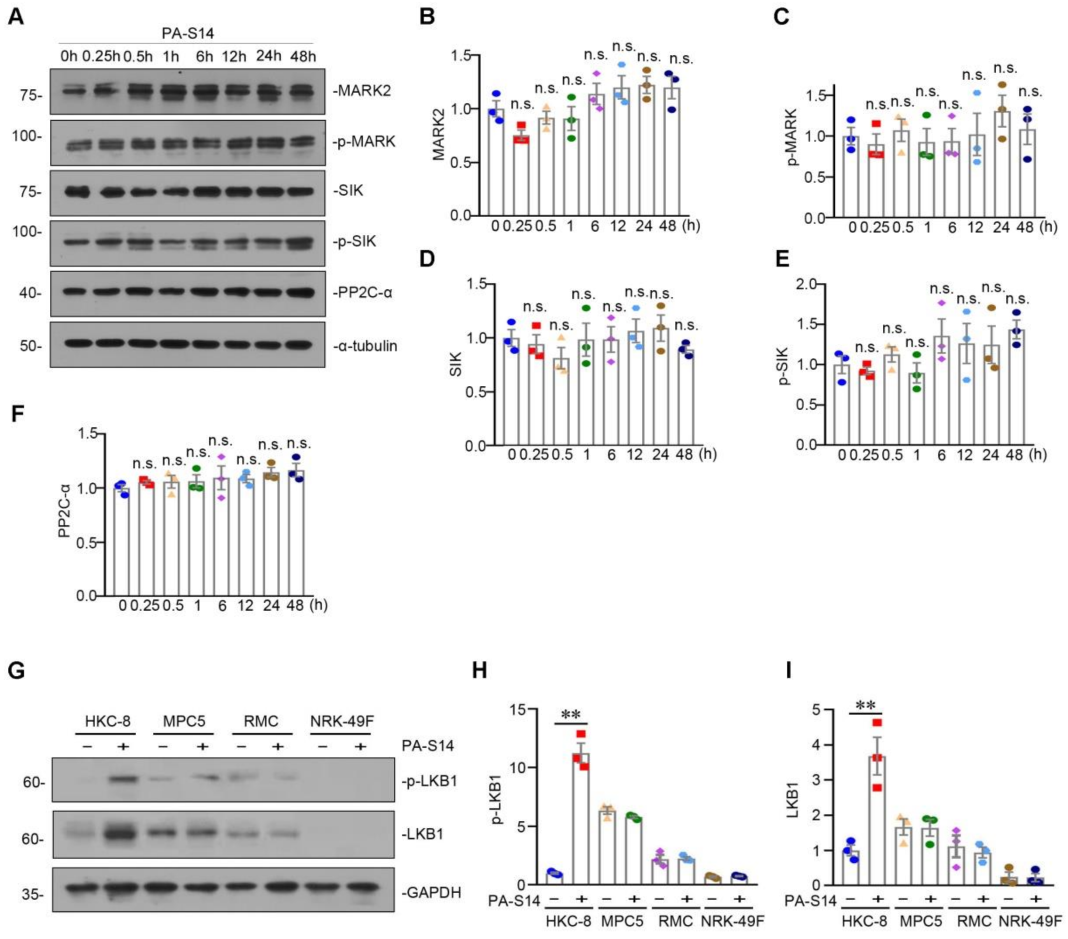

Supplementary Figure S8

Supplementary Figure S8. PA-S14 specifically took effects in renal tubular cells, and had no effects on other AMPK related kinases. (**A-F**) HKC-8 cells were treated with PA-S14 (0.5 μM) for indicated time period (0, 0.25, 0.5, 1, 6, 12, 24, 48 h). Representative western blot and quantitative data show the expression levels of MARK2, p-MARK, SIK, p-SIK and PP2C-α.(**G-I**) Representative western blot and quantitative data show the expression of p-LKB1 and LKB1 in tubular cell culture (HKC-8 cells), podocyte culture (MPC5 cells), mesangial cell culture (RMC cells), and fibroblast culture (NRK-49F cells). Different cells were treated with or without PA-S14 (0.5 μM) for 15 min. The whole cell lysates were blotted by antibodies

against p-LKB1 and LKB1. $^{**}P < 0.01$ versus control group.
